# Growth Kinetics, Carbon Isotope Fractionation, and Gene Expression in the Hyperthermophile *Methanocaldococcus jannaschii* during Hydrogen-Limited Growth and Interspecies Hydrogen Transfer

**DOI:** 10.1128/AEM.00180-19

**Published:** 2019-04-18

**Authors:** Begüm D. Topçuoğlu, Cem Meydan, Tran B. Nguyen, Susan Q. Lang, James F. Holden

**Affiliations:** a Department of Microbiology, University of Massachusetts, Amherst, Massachusetts, USA; b Institute for Computational Biomedicine, Weill Cornell Medical College, New York, New York, USA; c School of the Earth, Ocean, and Environment, University of South Carolina, Columbia, South Carolina, USA; North Carolina State University

**Keywords:** *Methanocaldococcus*, RNA-Seq, *Thermococcus*, carbon isotope fractionation, hydrogen, hyperthermophiles, methanogenesis, syntrophs

## Abstract

Hyperthermophilic methanogens are often H_2_ limited in hot subseafloor environments, and their survival may be due in part to physiological adaptations to low H_2_ conditions and interspecies H_2_ transfer. The hyperthermophilic methanogen Methanocaldococcus jannaschii was grown in monoculture at high (80 to 83 μM) and low (15 to 27 μM) aqueous H_2_ concentrations and in coculture with the hyperthermophilic H_2_ producer Thermococcus paralvinellae. The purpose was to measure changes in growth and CH_4_ production kinetics, CH_4_ fractionation, and gene expression in M. jannaschii with changes in H_2_ flux. Growth and cell-specific CH_4_ production rates of *M. jannaschii* decreased with decreasing H_2_ availability and decreased further in coculture. However, cell yield (cells produced per mole of CH_4_ produced) increased 6-fold when *M. jannaschii* was grown in coculture rather than monoculture. Relative to high H_2_ concentrations, isotopic fractionation of CO_2_ to CH_4_ (ε_CO2-CH4_) was 16‰ larger for cultures grown at low H_2_ concentrations and 45‰ and 56‰ larger for *M. jannaschii* growth in coculture on maltose and formate, respectively. Gene expression analyses showed H_2_-dependent methylene-tetrahydromethanopterin (H_4_MPT) dehydrogenase expression decreased and coenzyme F_420_-dependent methylene-H_4_MPT dehydrogenase expression increased with decreasing H_2_ availability and in coculture growth. In coculture, gene expression decreased for membrane-bound ATP synthase and hydrogenase. The results suggest that H_2_ availability significantly affects the CH_4_ and biomass production and CH_4_ fractionation by hyperthermophilic methanogens in their native habitats.

**IMPORTANCE** Hyperthermophilic methanogens and H_2_-producing heterotrophs are collocated in high-temperature subseafloor environments, such as petroleum reservoirs, mid-ocean ridge flanks, and hydrothermal vents. Abiotic flux of H_2_ can be very low in these environments, and there is a gap in our knowledge about the origin of CH_4_ in these habitats. In the hyperthermophile Methanocaldococcus jannaschii, growth yields increased as H_2_ flux, growth rates, and CH_4_ production rates decreased. The same trend was observed increasingly with interspecies H_2_ transfer between *M. jannaschii* and the hyperthermophilic H_2_ producer Thermococcus paralvinellae. With decreasing H_2_ availability, isotopic fractionation of carbon during methanogenesis increased, resulting in isotopically more negative CH_4_ with a concomitant decrease in H_2_-dependent methylene-tetrahydromethanopterin dehydrogenase gene expression and increase in F_420_-dependent methylene-tetrahydromethanopterin dehydrogenase gene expression. The significance of our research is in understanding the nature of hyperthermophilic interspecies H_2_ transfer and identifying biogeochemical and molecular markers for assessing the physiological state of methanogens and possible source of CH_4_ in natural environments.

## Abstract (precis)

Hyperthermophilic methanogens and H_2_-producing heterotrophs are collocated in high-temperature subseafloor environments, such as petroleum reservoirs, mid-ocean ridge flanks, and hydrothermal vents. Abiotic flux of H_2_ can be very low in these environments, and there is a gap in our knowledge about the origin of CH_4_ in these habitats. In the hyperthermophile Methanocaldococcus jannaschii, growth yields increased as H_2_ flux, growth rates, and CH_4_ production rates decreased. The same trend was observed increasingly with interspecies H_2_ transfer between *M. jannaschii* and the hyperthermophilic H_2_ producer Thermococcus paralvinellae. With decreasing H_2_ availability, isotopic fractionation of carbon during methanogenesis increased, resulting in isotopically more negative CH_4_ with a concomitant decrease in H_2_-dependent methylene-tetrahydromethanopterin dehydrogenase gene expression and increase in F_420_-dependent methylene-tetrahydromethanopterin dehydrogenase gene expression. The significance of our research is in understanding the nature of hyperthermophilic interspecies H_2_ transfer and identifying biogeochemical and molecular markers for assessing the physiological state of methanogens and possible source of CH_4_ in natural environments.

## INTRODUCTION

Each year, approximately 1 Pg of CH_4_ is produced globally through methanogenesis, largely by methanogens growing syntrophically with fermentative microbes that hydrolyze biopolymers (1), but little is known about the magnitude or mechanism of methanogenesis through thermophilic H_2_ syntrophy or interspecies H_2_ transfer. Deep-sea hydrothermal vents are known habitats for thermophilic methanogens (2). It was also estimated that 35% of all marine sediments are above 60°C (3), suggesting that these environments likewise provide a large global biotope for thermophiles. Microcosms containing low-temperature hydrothermal fluid as well as an archaeal coculture derived from a high-temperature oil pipeline each produced CH_4_ through interspecies H_2_ transfer at 80°C when supplemented with organic compounds, both without added H_2_ (4, 5). Both showed that CH_4_ was produced from a mixed microbial community consisting of the hyperthermophilic H_2_-producing heterotroph *Thermococcus* and the (hyper)thermophilic, hydrogenotrophic methanogens *Methanocaldococcus*, *Methanothermococcus*, and *Methanothermobacter*.

Molecular and culture-dependent analyses show that *Thermococcus* and thermophilic methanogens are collocated in hydrothermal vents (5–11), waters produced by high-temperature petroleum reservoirs (12–19), and mid-ocean ridge flanks (20). In high-temperature, organic-rich environments, such as petroleum reservoirs, collocated H_2_-producing heterotrophs are the primary source of H_2_ (21), but very little is known about this process at high temperatures or how thermophilic syntrophy affects environmental signals.

In this study, growth and CH_4_ production kinetics, carbon isotope fractionation, and gene expression data were examined together for a hyperthermophilic methanogen under conditions ranging from monoculture growth at high and low H_2_ concentrations to coculture growth with an H_2_-producing partner. The hyperthermophile Methanocaldococcus jannaschii was grown in monoculture in a chemostat under H_2_-replete and H_2_-limited conditions based on previous kinetic experiments (9). It was also grown with the H_2_-producing hyperthermophilic heterotroph Thermococcus paralvinellae using maltose and formate separately as the growth substrates (22). The purpose was to determine if *M. jannaschii* cell yield (amount of biomass produced per mole of CH_4_ produced, or *Y*_CH4_) increases when cultures are shifted from H_2_-replete to H_2_-limited growth conditions and if *Y*_CH4_ remains high or increases further during interspecies H_2_ transfer. This study also examined if interspecies H_2_ transfer stimulates the growth rate or cell yield of *T. paralvinellae* or ameliorates its H_2_ inhibition relative to its growth in monoculture. Furthermore, isotopic carbon fractionation was examined to determine if CH_4_ is isotopically lighter when H_2_ flux is reduced, as previously observed in moderately thermophilic methanogens (23–25). Finally, differential gene expression analysis using transcriptome sequencing (RNA-Seq) was used to determine if changes occur in *M. jannaschii* for the expression of genes for carbon assimilation, CH_4_ production, or energy generation when H_2_ decreases in availability. This study demonstrates the utility of measuring growth kinetic parameters, carbon isotope fractionation, and differential gene expression patterns for two species grown in coculture. The data elucidate how hyperthermophilic methanogens behave in a high H_2_ flux environment, such as those found at some hydrothermal vents, versus a low H_2_ flux environment, such as petroleum reservoirs.

## RESULTS

### Growth parameters for mono- and cocultures.

**FIG 1:**
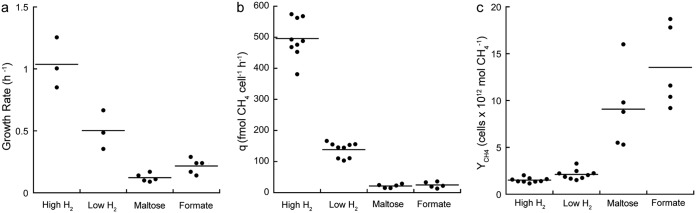
(a to c) Specific growth rate (a), cell-specific CH_4_ production rate (*q*) (b), and cell yield (*Y*_CH4_) (c) for *M. jannaschii* grown in monoculture in the chemostat with high (80 to 83 μM) and low (15 to 27 μM) aqueous H_2_ concentration and grown in coculture with *T. paralvinellae* in bottles using maltose and formate as growth substrates. The horizontal bar represents the mean value.

A summary of the growth conditions is provided in Table 1. In monoculture, the specific growth rate of *M. jannaschii* in the chemostat decreased from 1.04 ± 0.12 h^−1^ (± standard errors) when grown on 80 to 83 μM H_2_ to 0.50 ± 0.09 h^−1^ when grown on 15 to 27 μM H_2_ (Fig. 1A). Cell concentrations in the medium and H_2_ and CH_4_ concentrations in the headspace remained constant throughout growth in the chemostat (see Fig. S1 in the supplemental material). Attempts to grow *M. jannaschii* in coculture with *T. paralvinellae* in the chemostat when either maltose or formate was the energy source, with and without stirring and gas sparging of the medium with CO_2_ and N_2_, were unsuccessful. This was likely due to the open reactor that permits gas to flow out of the reactor without any gas pressure increase. Coculture growth was readily established in sealed bottles that contained 1 atm of gas pressure at room temperature. At 82°C, the gas pressure in the bottle was 1.2 atm, which slowed H_2_ efflux from the growth medium to the headspace. Therefore, the cocultures were grown in sealed bottles with the same volume of medium and headspace as the chemostat. The growth rates of *M. jannaschii* decreased further when it was grown in coculture with *T. paralvinellae* to 0.12 ± 0.01 h^−1^ and 0.22 ± 0.03 h^−1^ when *T. paralvinellae* was grown on maltose and formate, respectively (Fig. 1A). Relative to H_2_ concentrations when *T. paralvinellae* was grown in the bottles in monoculture, nearly all the H_2_ was removed from the coculture bottles and CH_4_ was produced (Fig. S2).

**TABLE 1:** Carbon isotopic composition of CO_2_ and CH_4_ of culture and coculture experiments

Growth condition	Initial H_2_ (aq^*c*^) (μM)	δ^13^C value (‰)			ε_CO2-CH4_ (‰)
		CO_2_ (aq)		CH_4_, *T_f_*	
		*T*_o_	*T_f_*		
*M. jannaschii* only					
Chemostat R1	83	−35.1	−29.0	−55.9	28.5
Chemostat R2	80	−34.6	−28.2	−55.9	29.3
Chemostat R3	80	−35.2	−28.4	−55.8	29.0
Chemostat R4	18	−35.9	−33.3	−75.7	45.9
Chemostat R5	15	−35.7	−31.8	−74.2	45.8
Chemostat R6	27	−35.8	−32.0	−72.5	43.7
Bottle B1	1,200^*a*^	−26.1	+22.6	−32.9	22.1^*b*^
Bottle B2	1,200^*a*^	−26.1	+19.2	−34.2	23.0^*b*^
*M. jannaschii-T. paralvinellae* coculture					
Bottle B3 (formate)	0	−26.7	−22.8	−99.4	85.1
Bottle B4 (formate)	0	−26.7	−23.0	−99.4	84.8
Bottle B5 (maltose)	0	−25.5	−24.4	−91.2	73.5
Bottle B6 (maltose)	0	−25.5	−21.6	−89.0	73.9

a Estimated at 82°C using the Geochemist’s Workbench Standard 10.0 (Aqueous Solutions, LLC, Champaign, Illinois, USA).

b Calculated based on the isotopic compositions of the starting CO_2_, final CO_2_, and accumulated methane.

c aq, aqueous concentration.

The cell-specific CH_4_ production rate decreased 3.6-fold when *M. jannaschii* was grown on 15 to 27 μM H_2_ (139 ± 8 fmol cell^−1^ h^−1^) relative to growth on 80 to 83 μM H_2_ (496 ± 21 fmol cell^−1^ h^−1^) (Fig. 1B). The rates decreased further when grown in coculture on maltose (21.3 ± 2.7 fmol cell^−1^ h^−1^) and on formate (24.8 ± 4.3 fmol cell^−1^ h^−1^) (Fig. 1B). However, the growth yields (*Y*_CH4_) for *M. jannaschii* grown in coculture were significantly higher when grown on maltose (9.1 ± 1.9 [×10^12^] cells per mol CH_4_) and formate (13.5 ± 2.0 [×10^12^] cells per mol CH_4_) than growth yields in monoculture on 15 to 27 μM H_2_ (2.1 ± 0.2 [×10^12^] cells per mol CH_4_) and 80 to 83 μM H_2_ (1.5 ± 0.1 [×10^12^] cells per mol CH_4_) (Fig. 1C). Summaries of the growth and CH_4_ production kinetics data for *M. jannaschii* are available in the supplemental material (Fig. S1 and S2 and Tables S1 and S2).

**FIG 2:**
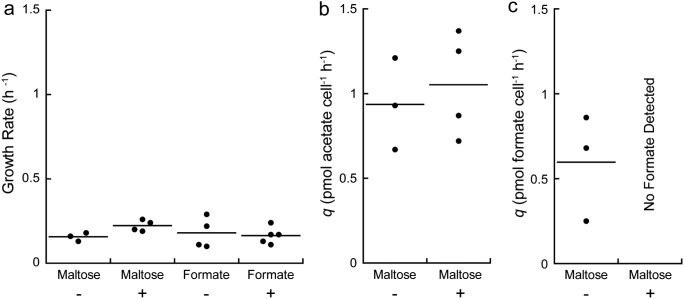
(a) Specific growth rate for *T. paralvinellae* grown in bottles in monoculture (−) and in coculture with *M. jannaschii* (+) on either maltose or formate. (b and c) Cell-specific production rate for acetate (b) and formate (c) for *T. paralvinellae* grown on maltose in monoculture (−) and in coculture with *M. jannaschii* (+). The horizontal bar represents the mean value.

There was no change in the specific growth rate or maximum cell concentration of *T. paralvinellae* when it was grown with or without *M. jannaschii* or with a change in carbon source (Fig. 2A and Fig. S2). The specific growth rates of *T. paralvinellae* grown on maltose in monoculture and in coculture were 0.16 ± 0.01 h^−1^ and 0.22 ± 0.02 h^−1^, respectively, while growth rates on formate in monoculture and in coculture were 0.18 ± 0.05 h^−1^ and 0.16 ± 0.02 h^−1^, respectively (Fig. 2A). Furthermore, when grown on maltose, there was no change in the growth yield (Table S3) or cell-specific acetate production rate of *T. paralvinellae* when grown in monoculture (0.94 ± 0.16 pmol cell^−1^ h^−1^) relative to growth in coculture (1.05 ± 0.15 pmol cell^−1^ h^−1^) (Fig. 2B). However, when grown on maltose, *T. paralvinellae* produced formate (in addition to H_2_ and acetate) when grown in monoculture (0.60 ± 0.18 pmol cell^−1^ h^−1^) but not when grown in coculture (Fig. 2C). The cell-specific H_2_ production rate was higher when *T. paralvinellae* was grown in monoculture on formate (130.9 ± 11.1 fmol cell^−1^ h^−1^) than for monoculture growth on maltose (0.9 ± 0.1 fmol cell^−1^ h^−1^) (Table S3). A summary of the growth and metabolite production kinetics data for *T. paralvinellae* is available in the supplemental material (Fig. S2 and Table S3). There was no growth of *M. jannaschii* when it was incubated in monoculture in medium supplemented with only 0.01% yeast extract or 0.1% sodium formate and 0.01% yeast extract with N_2_:CO_2_ in the headspace. These additions also did not stimulate the growth of *M. jannaschii* in monoculture when an H_2_:CO_2_ headspace was provided.

### Carbon isotope fractionation.

The final carbon isotopic composition (δ^13^C_CO2_) values were −24.4 to −21.6‰ in the coculture bottles and −33.3 to −28.2‰ in the chemostat (Table 1). The final δ^13^C_CO2_ values of the *M. jannaschii* monocultures in bottles were +19.2 to +22.6‰, demonstrating a substantial drawdown of the reactant. δ^13^C_CH4_ values became increasingly negative with increasing H_2_ limitation during cell growth. The δ^13^C_CH4_ values in the chemostat were −55.9 to −55.8‰ when *M. jannaschii* was grown on 80 to 83 μM H_2_ and decreased to −75.7 to −72.5‰ when grown on 15 to 27 μM H_2_. The corresponding values for isotopic fractionation of CO_2_ to CH_4_ (ε_CO2-CH4_) increased from 28.5 to 29.3‰ during high H_2_ growth to 43.7 to 45.9‰ during low H_2_ growth (Table 1). Similarly, δ^13^C_CH4_ values became more negative with increasing H_2_ limitation during coculture cell growth. In monoculture with 1.92 atm of initial H_2_ in the headspace at 82°C, the δ^13^C_CH4_ from *M. jannaschii* was −34.2 to −32.9‰ (Table 1). δ^13^C_CH4_ values decreased to −91.2 to −89.0‰ when *M. jannaschii* was grown in coculture with *T. paralvinellae* on maltose and to −99.4‰ when grown in coculture on formate. The corresponding ε_CO2-CH4_ values increased from 22.1 to 23.0‰ during monoculture growth in a serum bottle to 73.5 to 85.1‰ during growth in coculture with *T. paralvinellae* (Table 1).

### Transcriptomic analyses.

**FIG 3:**
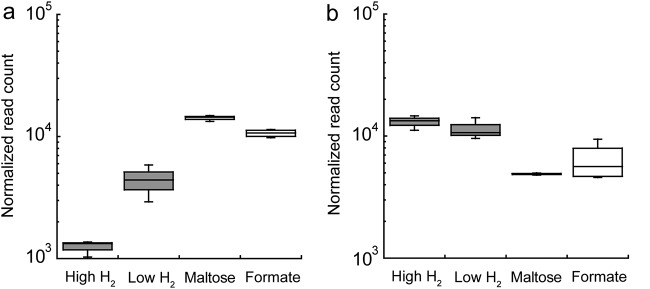
*M. jannaschii* transcript levels (relative log expression [RLE] normalization) for F_420_-dependent methylene-H_4_MPT dehydrogenase (*mtd*, MJ_RS05555) (a) and H_2_-dependent methylene-H_4_MPT I (*hmd*, MJ_RS04180) (b) for each growth condition.

RNA-Seq mapped 1,866 transcripts to the *M. jannaschii* genome. The thirteen samples that span four growth conditions were analyzed based on principal-component analysis (PCA) (Fig. S3A) and *t*-distributed stochastic neighbor embedding (*t*-SNE) (Fig. S3B) results. Pairwise comparisons of *M. jannaschii* grown in monoculture on high and low H_2_ showed up to 12 genes to be differentially expressed (adjusted *P* value of *<*0.01 and log_2_ fold change [|log_2_FC|] of >1) with 1 gene downregulated and 11 genes upregulated during growth on low H_2_ relative to growth on high H_2_ (Table S4). Under low-H_2_ conditions, F_420_-dependent methylene-tetrahydromethanopterin (H_4_MPT) dehydrogenase (*mtd*, MJ_RS0555 in the NCBI RefSeq database) gene expression increased 3.5-fold (Fig. 3A). There was no significant change in gene expression for H_2_-dependent methylene-H_4_MPT dehydrogenases (*hmd*, MJ_RS04180; *hmdX*, MJ_RS03820) (Fig. 3B and Fig. S4) or for any of the methyl-coenzyme M (CoM) reductase A I or II genes (*mcrA*, MJ_RS00415 and MJ_RS04540) (Fig. S5) for *M. jannaschii* grown in monoculture on high and low H_2_ in the chemostat. The genes that code for a GTP binding protein (MJ_RS01180), bacteriohemerythrin (MJ_RS03980), radical SAM protein (MJ_RS04390), a signal recognition particle (MJ_RS05550), a transcriptional regulator (MJ_RS06225), and four hypothetical proteins were upregulated on low H_2_, while a gene that codes for a histone (MJ_RS04990) was upregulated on high H_2_ (Table S4).

For cocultures grown on maltose, 97% of the reads mapped unambiguously to the *T. paralvinellae* genome and 1.5% mapped to the *M. jannaschii* genome. For cocultures grown on formate, 67% of the reads mapped unambiguously to the *T. paralvinellae* genome and 29% mapped to the *M. jannaschii* genome. These proportions generally matched the proportions of *T. paralvinellae* and *M. jannaschii* cells in each coculture type based on cell concentration estimates (Fig. S2). Merged pairwise comparisons of *M. jannaschii* gene expression for cultures grown in monoculture and *M. jannaschii* grown in coculture with *T. paralvinellae* showed up to 338 genes to be differentially expressed (adjusted *P* value of *<*0.01 and |log_2_FC| of >1) with 146 upregulated genes and 192 downregulated genes when grown in coculture relative to growth in monoculture on high and low H_2_ (Table S5). However, we cannot rule out the possibility that some of these gene expression changes are caused by the switch from the chemostat to bottles.

**FIG 4:**
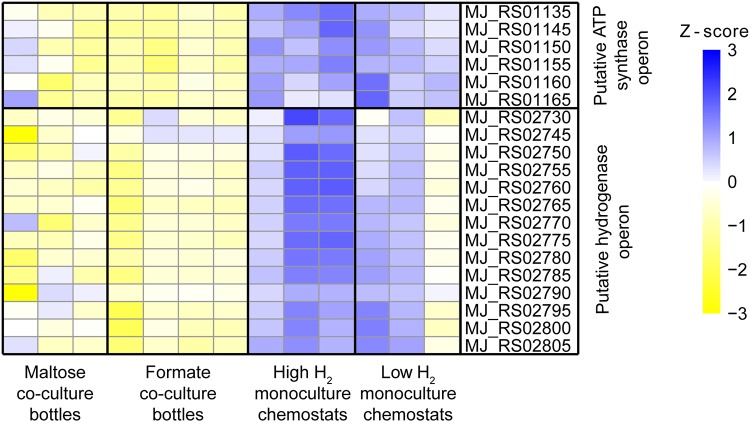
Differential gene expression analysis and RNA-Seq heat map for the *M. jannaschii* putative ATP synthase operon (MJ_RS01135 and MJ_RS01145 to MJ_RS01165) and the *M. jannaschii* putative hydrogenase operon (MJ_RS02730 and MJ_RS02745 to MJ_RS02805) for each growth condition.

F_420_-dependent methylene-H_4_MPT dehydrogenase (*mtd*, MJ_RS05555) gene expression was upregulated 4.3-fold in coculture relative to that of *M. jannaschii* grown under monoculture conditions (Fig. 3A). In contrast, gene expression of H_2_-dependent methylene-H_4_MPT dehydrogenases (*hmd*, MJ_RS04180; *hmdX*, MJ_RS03820) were both downregulated 2.1-fold in *M. jannaschii* grown in coculture relative to that of *M. jannaschii* grown in monoculture (Fig. 3B and Fig. S4). There was no change in gene expression for the methyl-CoM reductase I and II genes (Fig. S5). Gene expression for a hypothetical protein with a predicted RNA-binding domain (MJ_RS03480) showed a 22.5-fold increase in cocultures relative to monocultures (Fig. S6). Expression of 6 of the 9 *M. jannaschii* genes that code for a V-type ATP synthase (MJ_RS01130 to MJ_RS01165 and MJ_RS03255) were downregulated when cultures were grown in coculture relative to expression in *M. jannaschii* grown in monoculture (Fig. 4). Similarly, expression of 14 genes in a putative operon for membrane-bound, ferredoxin-dependent hydrogenase was also downregulated in *M. jannaschii* cultures grown in cocultures relative to cultures grown in monoculture (Fig. 4). These genes include Eha subunits A and B (MJ_RS02795 to MJ_RS02800), an oxidoreductase (MJ_RS02755), a dehydrogenase (MJ_RS02765), and a catalytic subunit (MJ_RS02730).

## DISCUSSION

Microorganisms in nature live in complex communities and biogeochemically impact their environment through interspecies metabolic interactions. Most of what is known about the kinetics and physiology of methanogenesis at various H_2_ concentrations and in coculture comes from studies of the thermophile Methanothermobacter thermoautotrophicus and the mesophile Methanococcus maripaludis. Growth rates of both organisms decreased when they were H_2_ limited relative to H_2_-replete growth. However, growth yields (*Y*_CH4_) increased when the cultures were H_2_ limited (26–28). Prior to this study, growth yields had not been measured for any methanogen during interspecies H_2_ transfer or for any hyperthermophilic methanogens under various H_2_ concentrations.

**FIG 5:**
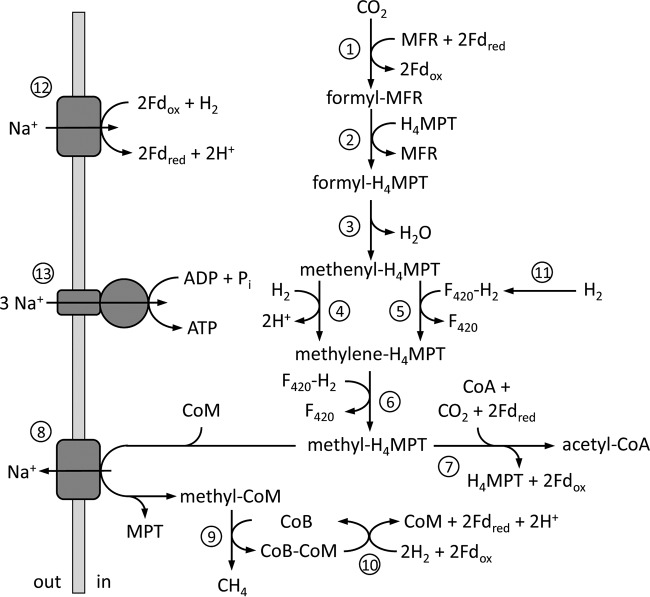
General metabolic pathway for *M. jannaschii*. The enzymes are (1) formylmethanofuran dehydrogenase, (2) formylmethanofuran:H_4_MPT formyltransferase, (3) cyclohydrolase, (4) H_2_-dependent methylene-H_4_MPT dehydrogenase (Hmd), (5) F_420_-dependent methylene-H_4_MPT dehydrogenase (Mtd), (6) methylene-H_4_MPT reductase (Mer), (7) CO dehydrogenase/acetyl-CoA synthase, (8) methyl-H_4_MPT:CoM methyltransferase, (9) methyl-CoM reductase (Mcr), (10) hydrogenase-heterodisulfide reductase complex, (11) F_420_-dependent hydrogenase, (12) membrane-bound ferredoxin-dependent hydrogenase, and (13) membrane-bound ATP synthase. MFR, methanofuran; H_4_MPT, tetrahydromethanopterin; F_420_, electron carrier coenzyme F_420_; CoA, coenzyme A; CoM, coenzyme M; CoB, coenzyme B; and Fd, electron carrier ferredoxin.

To determine *M. jannaschii* metabolism and kinetics under H_2_-replete and H_2_-limited growth conditions, as defined in a previous study (9), continuous growth in chemostats was established. The decrease in specific growth rate and cell-specific CH_4_ production rate of *M. jannaschii* when grown in monoculture under H_2_-limited conditions show that growth and methanogenesis rates are limited by H_2_ concentration. This trend continued when *M. jannaschii* was grown in coculture with *T. paralvinellae*, suggesting that interspecies H_2_ transfer led to further H_2_ limitation of methanogenesis. However, the cell yield for *M. jannaschii* increased when the cells were grown in coculture relative to growth in monoculture. This is consistent with previous studies that show higher cell yields for *M. thermoautotrophicus* and *M. maripaludis* upon H_2_ limitation, but there is no consensus on a physiological explanation (26–28). During methanogenesis, methyl-H_4_MPT is either converted to methyl-CoM for production of CH_4_ and energy generation on the cytoplasmic membrane or to acetyl-CoA for biosynthetic reactions (Fig. 5). Depending on the H_2_ concentration, hydrogenotrophic methanogens decide between maximum growth rate and maximum growth yield. This pattern can be explained by the rate-yield trade-off, which creates two divergent ecological strategies, namely, (i) slow growth but efficient metabolism and high yields when resources are scarce, and (ii) fast growth but inefficient metabolism and low yields upon rich resources. The rate-yield trade-off is suggested to be integral to evolution and the coexistence of species (29). It was proposed previously but not demonstrated that syntrophic growth of methanogens with a fermentative partner is optimized for cell yield rather that growth rate (27). In this study, *M. jannaschii* grew and produced CH_4_ solely on the H_2_ produced by *T. paralvinellae*, and the cell yield of *M. jannaschii* increased in coculture compared to that of growth in monoculture.

*Thermococcus* species use maltose for biosynthesis and energy generation that yields acetate and CO_2_ as well as H_2_ and a proton/sodium-motive force via a membrane-bound hydrogenase (30, 31). However, they are auxotrophic for certain amino acids that must be supplied from the environment (32, 33). *T. paralvinellae* increased gene expression of a membrane-bound formate hydrogenlyase operon and produced formate when inhibited by exogenous H_2_, suggesting that it converts H_2_ to formate when H_2_ is inhibited (22). *T. paralvinellae* also separately used formate as an energy source in the absence of maltose, produced H_2_, and generated a proton/sodium-motive force but required 0.01% yeast extract in the growth medium (22). Consequently, the cell-specific H_2_ production rate was ∼100-fold higher when cultures were grown on formate.

Morris et al. (34) defined microbial syntrophy as obligately mutualistic metabolism and included coculture growth between the hyperthermophilic H_2_ producer Pyrococcus furiosus and various hyperthermophilic methanogens, including *M. jannaschii*, as an example based on increased cell concentrations of both organisms in coculture relative to each in monoculture (35). Unlike *T. paralvinellae*, P. furiosus lacks formate hydrogenlyase as a mechanism to overcome H_2_ inhibition (36) and may be more dependent upon syntrophy to ameliorate H_2_ inhibition. In this study, when *T. paralvinellae* was grown with *M. jannaschii*, growth in coculture did not stimulate the growth rate, growth yield, or maximum cell concentration of *T. paralvinellae*. This suggests the relationship between *T. paralvinellae* and *M. jannaschii* is not obligately mutualistic and therefore more accurately represents interspecies H_2_ transfer rather than syntrophy. However, there was no formate production when *T. paralvinellae* was grown in coculture on maltose with *M. jannaschii*, and *M. jannaschii* cannot grow on formate (37 and this study), so *M. jannaschii* does appear to ameliorate H_2_ inhibition in *T. paralvinellae* when grown in coculture.

It was shown previously that the fractionation of carbon isotopes between CO_2_ and CH_4_ increased with decreasing concentrations of H_2_ availability or, more accurately, with decreasing Gibbs energy for the methanogenesis reaction (23). The ε_CO2-CH4_ fractionation factor for the thermophile Methanothermobacter marburgensis increased from 22 to 39‰ at high H_2_ concentrations to 58 to 64‰ at limiting H_2_ concentrations (23, 24). It was proposed that variations in the carbon isotopic fractionation factor are controlled by the extent of reversibility of the methanogenesis pathway, which was proposed to increase with decreasing Gibbs energy availability (23). In this study, the CH_4_ produced was isotopically more negative and the ε_CO2-CH4_ fractionation factor increased when *M. jannaschii* was grown in the chemostat with low H_2_ relative to high H_2_ conditions. Similarly, in bottles, CH_4_ was isotopically more negative and ε_CO2-CH4_ was much larger when *M. jannaschii* was grown in coculture with *T. paralvinellae* than when it was grown in monoculture with an initial estimated aqueous H_2_ concentration of 1.2 mM. The most negative CH_4_ in this study was produced when *M. jannaschii* was grown in coculture and H_2_ fluxes are presumably at their lowest rates.

Previous studies showed that during CO_2_ fixation and methanogenesis (Fig. 5) in *M. thermoautotrophicus* and *M. maripaludis*, gene expression for H_2_-dependent methylene-H_4_MPT dehydrogenase (*hmd*) decreased while expression of cofactor F_420_-dependent methylene-H_4_MPT (*mtd*) increased when growth was H_2_ limited relative to that of H_2_-replete growth (27, 28, 38). It was suggested that the Mtd reaction is the more reversible of the two methylene-H_4_MPT dehydrogenase reactions, which facilitates enhanced carbon isotope fractionation by methanogenesis pathway reversal in these methanogens under H_2_-limited conditions (23). The proteome of *M. jannaschii* contained a lower abundance of Hmd and higher abundances of Mtd and four flagellar proteins in early logarithmic growth phase when grown in batch phase under H_2_-limited conditions than under H_2_-replete conditions, but both Hmd and Mtd were found at high relative abundances in late logarithmic growth phase when grown under H_2_-replete conditions (39). During H_2_ syntrophy, the *M. thermoautotrophicus* proteome had more Mtd and less Hmd than were seen with monoculture growth under H_2_-replete conditions (40). There were no significant changes in gene expression or protein abundance for Hmd and Mtd in *M. maripaludis* during H_2_ syntrophy relative to that of an H_2_-limited monoculture (41).

In this study, RNA-Seq was used to determine changes in gene expression profiles in *M. jannaschii* for carbon assimilation, CH_4_ production, and energy generation pathways when there were changes in H_2_ availability. When *M. jannaschii* was grown under H_2_-limited conditions and in coculture, *mtd* expression was significantly upregulated and *hmd* expression was significantly downregulated in coculture cells compared to that of monoculture cells. This suggests a preference for F_420_ as an electron carrier in the methanogenesis pathway under H_2_-limited conditions. The increase in cell yield in coculture was not supported by a change in the expression of genes in the carbon assimilation and methanogenesis pathways. No significant changes were detected in the expression of methyl-CoM reductase I and II and methyl-H_4_MPT:CoM methyltransferase, which catalyze the last two steps of methanogenesis (Fig. 5). Previously, changes in the relative abundances of methyl-CoM reductases I and II were observed in *M. thermoautotrophicus* with H_2_ availability and growth during syntrophy (27, 40, 42). Moreover, there was no change in expression in our study in the carbon monoxide dehydrogenase/acetyl-CoA synthase genes, which code for the enzyme that converts methyl-MPT to acetyl-CoA.

In coculture, there was up to a 22.5-fold increase in the expression of a putative RNA binding protein that is only found in methanogens and the *Thermococcales* and has been proposed to regulate cellular activity at the translation level (43). The decrease in the expression of genes in the putative membrane-bound, ferredoxin-dependent hydrogenase operon and in the membrane-bound, Na^+^-translocating V-type ATPase operon supports the kinetic observations that *M. jannaschii* is energy limited when grown in coculture. Under H_2_-limited coculture conditions, the cell must direct more of its methyl-H_4_MPT toward biosynthesis. Furthermore, there was no change in the expression of the genes for flagella. This was different from what was previously observed for *M. jannaschii* using proteomics (39) and may be due to the use of a chemostat in this study instead of a batch reactor.

In environments such as low-H_2_ hydrothermal vents along subduction zones and some mid-ocean ridges, oil reservoirs, and high saline shale beds where organic compounds are present and H_2_ efflux rates are low, thermophilic methanogens like *M. jannaschii* likely can grow and produce CH_4_ through interspecies H_2_ transfer with hyperthermophilic H_2_-producing heterotrophs, like *T. paralvinellae*, with high cell yields and large carbon isotope fractionations, but they do so at very low rates. This likely explains the presence of thermophilic H_2_ producers and thermophilic, hydrogenotrophic methanogens in petroleum reservoirs and may be a source of CH_4_ in that habitat. In contrast, high-temperature methanogens in high-H_2_ hydrothermal vents, such as those supported by serpentinization and following volcanic eruptions (2), may subsist entirely from abiotic H_2_ with elevated cell-specific CH_4_ production rates and smaller carbon isotope fractionations. Metatranscriptomic analyses coupled with carbon isotope analyses of native CH_4_ will help to determine what fraction of methanogenesis in a high-temperature environment is due to interspecies H_2_ transfer relative to growth on abiotic H_2_. In this manner, we will be better equipped to model cooperative, competitive, and neutral interactions between different species in an environment and predict the biogeochemical outcome of a mixed community living in a habitat.

## MATERIALS AND METHODS

### Growth media and culture conditions.

Methanocaldococcus jannaschii DSM 2661 (37) and Thermococcus paralvinellae DSM 27261 (44) were purchased from the Deutsche Sammlung von Mikroorganismen und Zellkulturen (DSMZ). The growth medium for pure cultures of *M. jannaschii* was based on DSM medium 282 (9). For the cocultures of *M. jannaschii* and *T. paralvinellae* and monoculture of *T. paralvinellae,* the base medium was amended with 0.01% (wt vol^−1^) yeast extract (vitamin B_12_ fortified; Difco), 1 μM Na_2_WO_4_·2H_2_O, 0.26 μM (NH_4_)_2_Fe(SO_4_)_2_·6H_2_O, and 0.25 μM (NH_4_)_2_Ni(SO_4_)_2_·6H_2_O. The primary carbon and energy source added for *T. paralvinellae* was either 0.5% (wt vol^−1^) maltose (Sigma) or 0.1% (wt vol^−1^) sodium formate (Fluka). All media were pH balanced to 6.00 ± 0.05 and reduced with 0.025% (wt vol^−1^) each of cysteine-HCl and Na_2_S·9H_2_O before inoculation. To test if *M. jannaschii* can use formate or yeast extract for growth in the absence of H_2_, or if they stimulate growth in the presence of H_2_, *M. jannaschii* was incubated in monoculture on the base medium amended with 0.1% formate and 0.01% yeast extract or 0.01% yeast extract only as described above, each in serum bottles with 1 additional atm (100 kPa) of either H_2_:CO_2_ (80%:20%) or N_2_:CO_2_ (80%:20%) added to the headspace at room temperature prior to incubation.

*M. jannaschii* was grown in monoculture at 82°C and under high and low H_2_ concentrations in a chemostat to measure its growth and CH_4_ production kinetics and to generate biomass for gene expression analysis. A 2-liter bioreactor (all-in-one benchtop reactor; Ace Glass) with gas flow, temperature (±0.1°C), and pH (±0.1 unit; Eutech Instruments pH 200 Series) controls was used with 1.5 liters of growth medium. The medium was maintained at pH 6.0 ± 0.1 by the automatic addition of 0.25 mM HCl. For high-H_2_ conditions, the bioreactor was gassed with a mixture of CO_2_ (20.5 ml min^−1^) and H_2_ (132 ml min^−1^). For low-H_2_ conditions, the bioreactor was gassed with a mixture of CO_2_ (20.5 ml min^−1^), N_2_ (130 ml of gas min^−1^), and H_2_ (2.5 ml min^−1^). Pure gases were blended using a mass flow controller (Matheson Tri-Gas) and added to the bioreactor through a single submerged fritted bubbler (70 to 100 μm; Ace Glass; ASTM certified). The reactor is an open system and remains at ambient gas pressure. It was stirred at 150 to 180 rpm using a four-blade open impeller (6-cm diameter) with a glass shaft and Teflon blades. Aqueous H_2_ and CH_4_ concentrations were measured before and after inoculation by drawing 25 ml of medium from the bottom of the bioreactor directly into anoxic 60-ml serum bottles and measuring the headspace gas. H_2_ was measured using a gas chromatograph fitted with a thermal conductivity detector (Shimadzu GC-8A) and a 60/80 Carboxen 1000 column (15 feet by 1/8 inch; Supelco). CH_4_ was measured using a gas chromatograph fitted with a flame ionization detector (Shimadzu GC-17A) and a 5A 80/100 molecular sieve column (6 feet by 1/8 inch; Alltech). The aqueous H_2_ concentrations in the bioreactor prior to inoculation were 80 to 83 μM for the high-H_2_ condition and 15 to 27 μM for the low-H_2_ condition (Table 1).

The media were inoculated with 50 to 100 ml of a logarithmic-growth-phase culture of *M. jannaschii*. During growth, liquid samples were drawn from the bioreactor and cell concentrations were determined using phase-contrast light microscopy and a Petroff-Hausser counting chamber. The growth rate (*k*) was determined by plotting cell concentration against time and fitting a logarithmic curve to the growth data. *M. jannaschii* was grown in batch reactor mode until the culture reached mid-logarithmic growth phase, and then the bioreactor was switched to chemostat mode by pumping sterile growth medium into the bioreactor from a sealed 12-liter reservoir that was degassed with N_2_ through a submerged glass tube and heated to 75°C. Simultaneously and at the same rate, spent growth medium was pumped out of the bioreactor using a dual-channel peristaltic pump. The H_2_ and CH_4_ concentrations in the headspace of the bioreactor were measured using gas chromatography as described above. At high and low H_2_ concentrations, cells were grown in the reactor at low enough cell concentrations such that there was excess H_2_ in the headspace and the cells were not H_2_ limited (see Fig. S1 in the supplemental material).

Growth of *M. jannaschii* was stable in the chemostat after three volume replacements of the medium within the reactor (∼5 h for high H_2_, ∼14 h for low H_2_) and was monitored for an additional ∼0.5 volume replacements to obtain kinetic data. The CH_4_ production rate per cell (*q*) was calculated from the sum of the CH_4_ concentration in the headspace times the gas flow rate and the CH_4_ concentration in the medium times the medium dilution rate (i.e., CH_4_ production rate), which was normalized by the total cell concentration in the reactor. The cell yield per mole of CH_4_ produced (*Y*_CH4_) was calculated by dividing the cell production rate (dilution rate times cell concentration) by the CH_4_ production rate. The complete contents of the bioreactor then were drained into ice-cooled centrifuge bottles, spun in a centrifuge at 10,000 × *g* and 4°C for 60 min, resuspended in 1 ml of TRIzol (Invitrogen), and frozen at −80°C until processed. Chemostats were run in triplicate for both conditions.

*M. jannaschii* and *T. paralvinellae* were grown in coculture at 82°C in 2-liter gas-tight flasks (Pyrex bottles sealed with rubber lyophilization stoppers) containing 1.5 liters of medium with ambient pressure of N_2_:CO_2_ (80%:20%) in the headspace at room temperature without agitation and either maltose or formate as the energy source (Table 1). Separate logarithmic-growth-phase cultures of *M. jannaschii* and *T. paralvinellae* were combined to inoculate the bottles. The coculture was established immediately and did not require prior coculture transfers. At various times during growth, total cell concentration in bottles was determined using a Petroff-Hausser counting chamber and phase-contrast light microscopy. The *M. jannaschii* cell concentration was determined by counting the number of autofluorescent cells using epifluorescence microscopy and UV light excitation (45). The concentration of *T. paralvinellae* cells was calculated by subtracting the concentration of *M. jannaschii* cells from the total cell concentration. The pH change was <0.1 pH units during growth. For comparison, *T. paralvinellae* was grown separately in the same bottles and conditions in monoculture on 0.5% maltose and separately on 0.1% sodium formate, both with ambient pressure of N_2_:CO_2_ in the headspace at room temperature. Cell concentrations were measured as described above.

The growth rates (*k*) of *M. jannaschii* and *T. paralvinellae* were determined by plotting cell concentration against time and fitting a logarithmic curve to the growth data. The total amounts of CH_4_ and H_2_ in the bottles were determined by gas chromatography. The concentrations of formate, acetate, butyrate, isovalerate, and 2-methylbutyrate were measured from aliquots of syringe-filtered (0.2-μm pore size) spent medium from each coculture and *T. paralvinellae* monoculture incubation at various time points (for maltose growth only) using ultra-high-pressure liquid chromatography (UHPLC) as previously described (46). Methanogen cell yields (*Y*_CH4_) were determined from the linear slope of the number of methanogen cells per bottle plotted against the amount of CH_4_ per bottle (47). The rate of CH_4_ production per cell is calculated from *k*/(0.693 × *Y*_CH4_) as previously described (47). Similarly, *T. paralvinellae* cell yields based on acetate and formate produced and for H_2_ produced (for monoculture only) were determined from the linear slope of *T. paralvinellae* cell concentration plotted against acetate, formate, or H_2_ concentration. When the cocultures reached late logarithmic growth phase, the cells were harvested for transcriptome analysis as described above (*T. paralvinellae* cells were not harvested when grown in monoculture). Cocultures grown on maltose were grown in triplicate, while cocultures grown on formate were grown in quadruplicate.

### Carbon isotope fractionation.

At the start (*T_o_*) and end (*T_f_*) of each chemostat run, 20 ml of chemostat headspace was transferred in triplicate into evacuated vials (Labco Exetainer). *M. jannaschii* also was grown in monoculture in 245-ml serum bottles containing 100 ml of medium and 1 additional atm (100 kPa) of H_2_:CO_2_ (80%:20%) added to the headspace at room temperature prior to incubation. *M. jannaschii* was also grown in coculture with *T. paralvinellae* in 245-ml serum bottles containing 100 ml of either 0.5% maltose medium or 0.1% sodium formate medium as described above. The isotopic signatures of CH_4_ were determined using a gas chromatography-combustion-isotope ratio mass spectrometer (GC-C-IRMS; Thermo Scientific) equipped with a GS-CarbonPlot column (30 m long, 0.320-mm inner diameter, 1.50-μm film thickness; Agilent). Isotopic signatures were determined using external CH_4_ standards of known isotopic signatures (−57.40 ± 0.06‰) that were obtained from Arndt Schimmelmann (Indiana University). The error of the analysis was determined from external standards, and the standard deviation of multiple injections was 0.3‰. At *T_o_* and *T_f_* of the chemostat runs and the serum bottles, triplicate samples of dissolved inorganic carbon (DIC) were drawn from the growth medium. Each DIC sample (either 0.8 or 1.0 ml) was syringe filtered (0.2 μm pore size) and injected into prepared vials (Labco Exetainer) that had been flushed with He and contained 100 μl of phosphoric acid. Samples were analyzed by GasBench-IRMS. DIC standards were prepared in concentrations from 0.5 to 7.0 mM using KHCO_3_ and Li_2_CO_3_ of known isotopic composition (−38.1‰ and −1.1‰, respectively). The error of analysis was determined from external standards, and the standard deviation of multiple injections was 0.3‰. The δ^13^C_CO2_ value was calculated from the δ^13^C_DIC_ value using the relationship of Mook et al. (48) at the temperature of the cultures (82°C).

Carbon isotopic compositions are presented as δ^13^C in the per mille notation (‰) relative to the VPDB (Vienna Pee Dee Belemnite) standard:

(1) $${\text{δ}}^{13}\text{C}=\left[\frac{R_{\text{Sample}}}{\;R_{\text{Standard}}}\right]-1\times 10^{3}\;\left(‰\right)$$

where *R*_sample_ is the ^13^C/^12^C ratio of the sample and *R*_standard_ is 0.0112372. The ε notation is used to express isotope fractionation factors in per mille (‰):

(2) $${\text{ε}}_{\mathrm{CO}2-\mathrm{CH}4}=\left({\text{α}}_{\mathrm{CO}2-\text{CH}4}-1\right)\;\times 10^{3}\;\left(‰\right)$$

The fractionation factor, α, is defined as the ratio between the isotopic ratio in the substrate and product:

(3) $${\text{α}}_{\text{CO}2-\mathrm{CH}4}=\frac{R_{\mathrm{CO}2}}{R_{\mathrm{CH}4}}=\frac{{\text{δ}}^{13}{\text{C}}_{\mathrm{CO}2}+10^{3}}{{\text{δ}}^{13}{\text{C}}_{\mathrm{CH}4}+10^{3}}$$

where *R*_CO2_ is the ^13^C/^12^C ratio of the initial CO_2_ and *R*_CH4_ is the ^13^C/^12^C ratio of the CH_4_ produced. The propagated error of the fractionation factors was 0.4‰, except in the case of the *M. jannaschii* monoculture.

The inorganic carbon in the *M. jannaschii* monoculture serum bottles was extensively drawn down, substantially altering the ^13^C signature of the remaining reactant. The fractionation factor was therefore calculated by setting the initial CO_2_ isotopic signature equal to that in serum bottles without cells (−26.1 ± 0.8‰) and reacting it stepwise under different fractionation factors. To obtain final isotopic compositions that match the remaining CO_2_ (+18.9 and +15.5‰) and the final accumulated product, CH_4_ (−32.9‰ and −34.2‰), fractionation factors of 22.1 ± 1.3‰ and 23.0 ± 1.3‰ were required in the two different experiments.

### RNA-Seq analysis.

Total RNA was extracted from 13 cell pellets from each growth condition (Table 1) using a Direct-zol RNA extraction kit (Zymo). RNA quantity was determined using Qubit fluorometry. RNA integrity was checked using an Agilent 2100 bioanalyzer, a NanoDrop 2000 spectrophotometer, and gel electrophoresis of the RNA, followed by staining with ethidium bromide. Removal of rRNA, library construction, multiplexing, and sequencing of the mRNA using an Illumina HiSeq2500 sequencer with two 150-bp paired ends was performed commercially by GENEWIZ, LLC (South Plainfield, NJ, USA), as described by the company. Sequencing depths ranged from 30,751,946 to 41,634,527 sequence reads per sample, with a median of 34,532,231 and a mean of 35,155,474 reads per sample. The RNA-Seq reads were mapped to both *M. jannaschii* and *T. paralvinellae* genomes using BBSplit from the BBMap package (https://sourceforge.net/projects/bbmap/). BBSplit is an aligner tool that bins sequencing reads by mapping them to multiple references simultaneously and separates the reads that map to multiple references to a special “ambiguous” file for each of them. For further analyses, we removed all ambiguously mapped reads to both genomes and worked with only the reads that unambiguously map to the *M. jannaschii* genome. Two to 5% of the reads were lost in this step.

The mapped reads for *M. jannaschii* were aligned to the *M. jannaschii* genome and sorted using the STAR aligner, version 2.5.1b (49). Aligned sequence reads were assigned to genomic features and quantified using the featureCounts read summarization tool (50). The output of the analyses generated BAM files containing the sequence of every mapped read and its mapped location. An unsupervised *t*-SNE algorithm (51) and PCA were used to predict outliers among the total RNA sample replicates.

Genes that were differentially expressed were identified using DESeq2 in the Bioconductor software framework (https://www.bioconductor.org) in R (version 3.3 [http://www.r-project.org]) and on a Galaxy platform using DEBrowser (52–55). Relative log expression normalization was performed by using the R package DESeq2. The DESeq2 package allows for sequencing depth normalization between samples, estimates gene-wise dispersion across all samples, fits a negative binomial generalized linear model, and applies Wald statistics to each gene. The genes were reported as differentially regulated if the |log_2_FC| value was >1 and the adjusted *P* value was <0.01. Heatmaps were plotted in R (version 3.3 [http://www.r-project.org]) using the pheatmap package. The heatmap color scale represents the z-score, which is the number of standard deviations the mean score of the treatment is from the mean score of the entire population.

### Data availability.

The count files and raw sequences are available in the NCBI Gene Expression Omnibus (GEO) database under accession no. GSE112986.

## Supplementary Material

Supplemental file 1
