# Supplementary material for: Growth Kinetics, Carbon Isotope Fractionation, and Gene Expression in the Hyperthermophile Methanocaldococcus jannaschii during Hydrogen-Limited Growth and Interspecies Hydrogen Transfer
Source: Appl Environ Microbiol. 2019 Apr 18;85(9):e00180-19. doi: 10.1128/AEM.00180-19 (PMC6495749; doi:10.1128/AEM.00180-19)
Supplement: Supplemental file 1 [file AEM.00180-19-s0001.pdf]

Supplemental Material for

Growth Kinetics, Carbon Isotope Fractionation and Gene Expression in the Hyperthermophile *Methanocaldococcus jannaschii* during H_2-Limited Growth and Interspecies H_2 Transfer

Begüm D. Topçuoğlu[a*], Cem Meydan[b], Tran B. Nguyen[c], Susan Q. Lang[c], and James F. Holden[a#]

[a]*Department of Microbiology, University of Massachusetts, Amherst, Massachusetts, USA.*
[b]*Institute for Computational Biomedicine, Weill Cornell Medical College, New York, New York, USA*
[c]*School of the Earth, Ocean, and Environment, University of South Carolina, Columbia, South Carolina, USA*

#Address correspondence to James F. Holden, jholden@microbio.umass.edu
*Present address: Begüm D. Topçuoğlu, Department of Microbiology & Immunology, University of Michigan, Ann Arbor, Michigan.

This file includes:

Figures S1 through S6
Tables S1 through S5

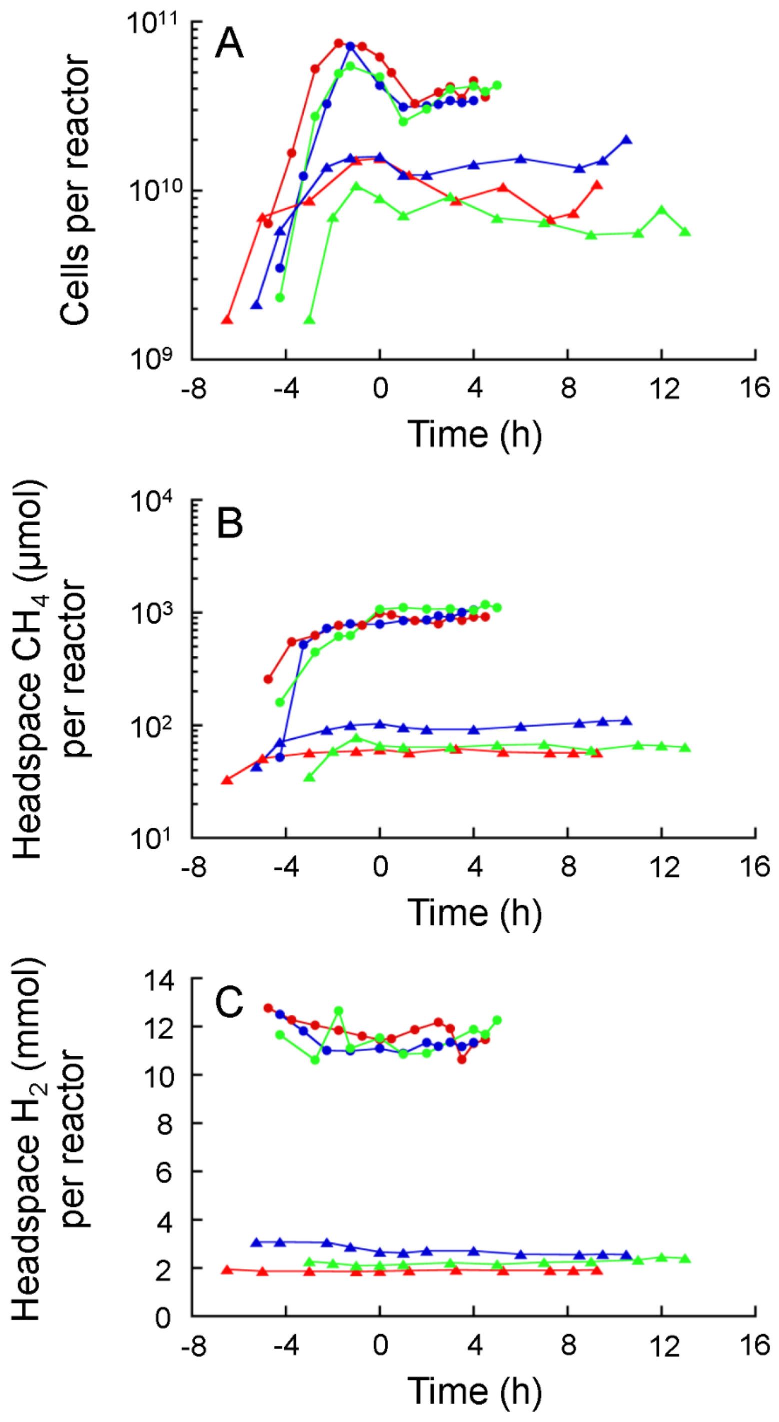

Figure S1 Total number of cells in the medium (a) and total amount of CH_4 (b) and H_2 (c) in the reactor headspace for *M. jannaschii* grown in the chemostat at 80-83 µM H_2 (●, ●, ●) and 15-27 µM H_2 (▲, ▲, ▲). Time zero indicates the point where the reactor was shifted from batch growth to chemostat growth.

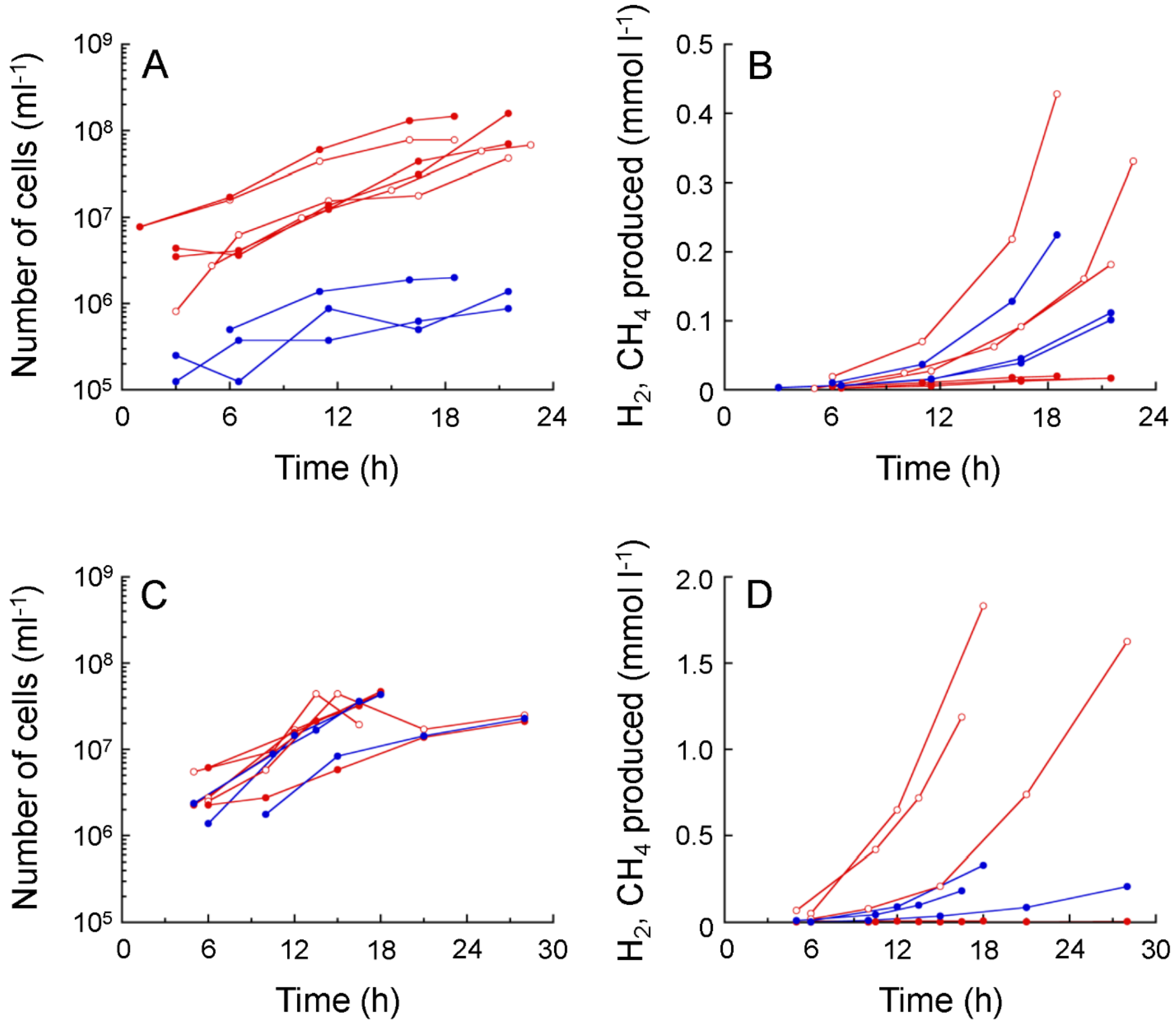

Figure S2 Cell concentrations and H_2 and CH_4 produced per liter of growth medium when cultures were grown in bottles on maltose (a, b) and formate (c, d). Symbols: (○) cell concentration of *T. paralvinellae* (a, c) and H_2 produced (b, d) when *T. paralvinellae* was grown in monoculture, (●) cell concentration of *T. paralvinellae* (a, c) and H_2 produced (b, d) when *T. paralvinellae* and *M. jannaschii* were grown in co-culture, (●) cell concentration of *M. jannaschii* (a, c) and CH_4 produced (b, d) when *T. paralvinellae* and *M. jannaschii* were grown in co-culture.

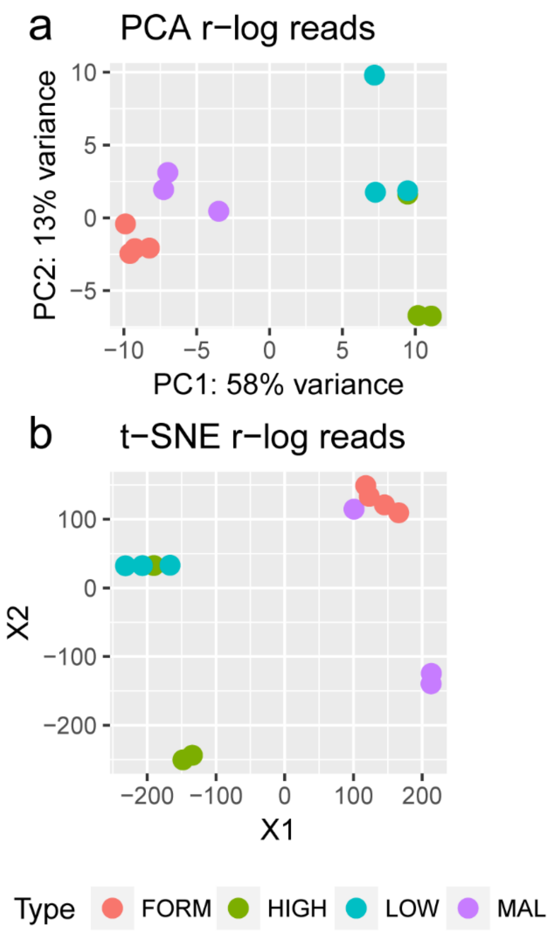

Figure S3 (a) Principal component analysis (PCA) (b) t-Distributed Stochastic Neighbor Embedding Analysis (t-SNE). Red and purple represent co-cultures on formate and maltose, respectively. Blue and green represent chemostat monocultures with 15-27 μM H_2 and 80-83 μM H_2 conditions, respectively.

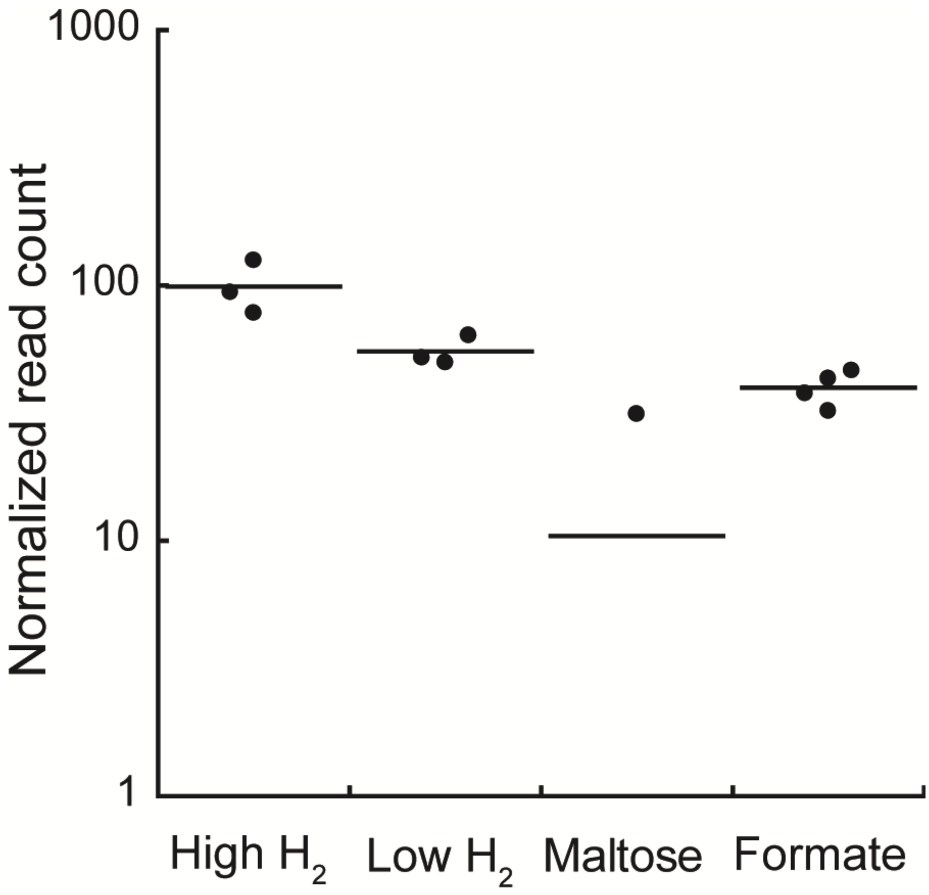

Figure S4 *M. jannaschii* transcript levels (relative log expression (RLE)-normalization) for H_2-dependent methylene-H_4MPT (*hmd*X. MJ_RS03820) for each growth condition.

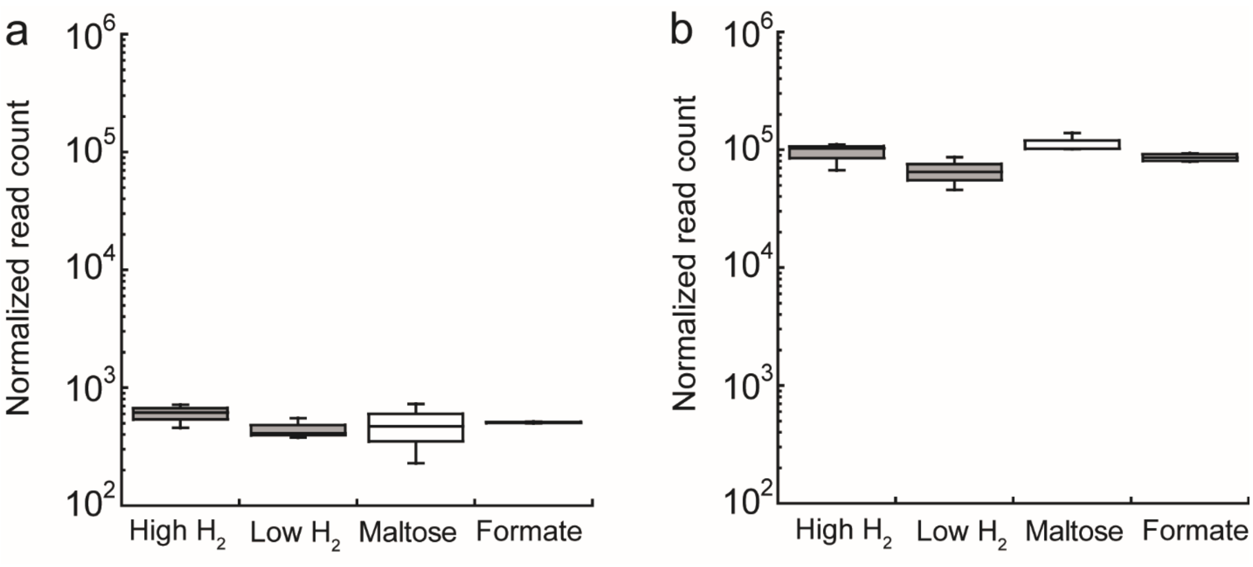

Figure S5 *M. jannaschii* transcript levels (relative log expression (RLE)-normalization) for (a) methyl-CoA reductase I subunit A (*mcrA*, MJ_RS00415) (b) methyl-CoA reductase II subunit A (*mcrA*, MJ_RS04540) for each growth condition.

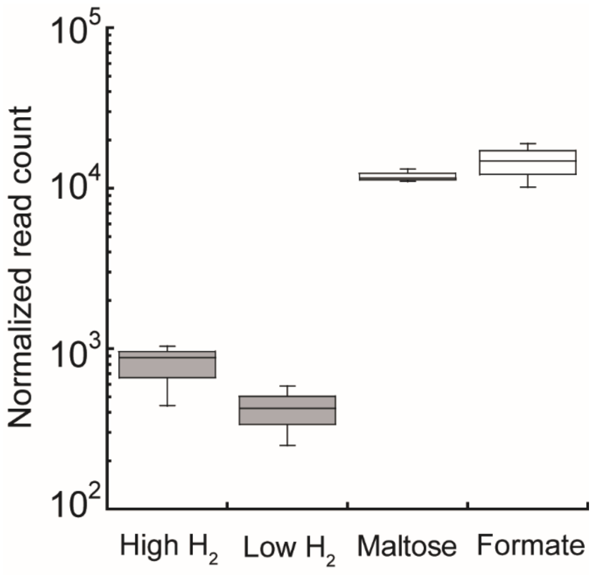

Figure S6 *M. jannaschii* transcript levels (relative log expression (RLE)-normalization) for MJ_RS03480, a hypothetical protein with a predicted RNA-binding domain.

Table S1. Chemostat data for *Methanocaldococcus jannaschii*

$[H_2]$ (µM)	D (h^{-1})	Dilution rate (ml min^{-1})	[Cell] (ml^{-1})	Cell prod. rate (cells h^{-1})	Liquid [CH_4] (nmol ml^{-1})	Headspace [CH_4] (µmol ml^{-1})	CH_4 prod. rate (mmol h^{-1})	Specific CH_4 prod. rate (fmol $cell^{-1}$ h^{-1})	Cell yield (cells × 10^{12} mol CH_4^{-1})
80	0.8	20	2.26×10^{7}	2.72×10^{10}	30.2	1.899	15.87	453.0	1.71
80	0.8	20	2.88×10^{7}	3.46×10^{10}	30.2	2.036	17.02	381.2	2.03
80	0.8	20	2.31×10^{7}	2.77×10^{10}	30.2	2.040	17.05	476.2	1.62
80	0.8	20	2.20×10^{7}	2.64×10^{10}	44.1	2.009	16.81	492.9	1.57
80	0.8	20	2.14×10^{7}	2.57×10^{10}	44.1	2.231	18.66	562.5	1.38
80	0.8	20	2.20×10^{7}	2.64×10^{10}	44.1	2.342	19.59	574.3	1.35
83	0.68	17	2.68×10^{7}	2.73×10^{10}	34.3	2.329	19.46	468.4	1.40
83	0.68	17	2.49×10^{7}	2.54×10^{10}	34.3	2.624	21.92	567.9	1.16
83	0.68	17	2.71×10^{7}	2.77×10^{10}	34.3	2.454	20.50	488.1	1.35
17	0.34	8.6	4.38×10^{6}	2.26×10^{9}	3.2	0.128	1.1	166.5	2.05
17	0.34	8.6	4.75×10^{6}	2.45×10^{9}	3.2	0.128	1.1	153.6	2.23
17	0.34	8.6	7.00×10^{6}	3.61×10^{9}	3.2	0.127	1.1	103.3	3.28
22	0.28	7.0	8.75×10^{6}	3.68×10^{9}	0.1	0.234	2.1	155.3	1.75
22	0.28	7.0	9.75×10^{6}	4.10×10^{9}	0.1	0.243	2.2	144.7	1.86
22	0.28	7.0	1.30×10^{7}	5.46×10^{9}	0.1	0.248	2.2	110.8	2.48
28	0.25	6.3	5.63×10^{6}	2.13×10^{9}	5.5	0.151	1.4	156.0	1.52
28	0.25	6.3	7.75×10^{6}	2.93×10^{9}	5.5	0.147	1.3	110.3	2.25
28	0.25	6.3	5.75×10^{6}	2.17×10^{9}	5.5	0.144	1.3	145.6	1.67

D = (Dilution rate × 60) ÷ 1,550 ml
Cell production rate = Dilution rate × [Cell] × 60
CH_4 production rate = ((Liquid [CH_4] × Dilution rate) + (Headspace [CH_4] × Gas flow rate)) × 60
Cell-specific CH_4 production rate (q) = CH_4 production rate ÷ ([Cell] × 1,500 ml)
Cell yield (Y_{CH4}) = Cell production rate ÷ CH_4 production rate

Table S2. Co-culture growth and CH_4 production kinetic data for *Methanocaldococcus jannaschii*

Growth condition	Growth rate (k, h^{-1})	Yield (Y_{CH4}) (10^{12} cells mol CH_4^{-1})	CH_4 prod. rate (fmol CH_4 $cell^{-1}$ h^{-1})
Maltose	0.11	5.5	28.9
Maltose	0.10	9.8	14.7
Maltose	0.09	5.3	24.5
Maltose	0.14	8.8	23.0
Maltose	0.17	16.0	15.3
Formate	0.24	10.4	33.3
Formate	0.29	11.6	36.1
Formate	0.24	17.8	19.4
Formate	0.14	9.2	22.0
Formate	0.17	18.7	13.1

Cell-specific CH_4 production rate (q) = (k/[0.693 × Y_{CH4}])

Table S3. Growth metabolite production kinetic data for *Thermococcus paralvinellae*

Growth condition	Growth rate (k, h^{-1})	Yield (Y_{H2}) (10^{12} cells mol H_2^{-1})	H_2 prod. rate (fmol H_2 $cell^{-1}$ h^{-1})[a]	Yield ($Y_{acetate}$) (10^{12} cells mol $acetate^{-1}$)	Acetate prod. rate (pmol acetate $cell^{-1}$ h^{-1})	Yield ($Y_{formate}$) (10^{12} cells mol $formate^{-1}$)	Formate prod. rate (pmol formate $cell^{-1}$ h^{-1})
Monoculture:							
Maltose	0.16	286	0.81	0.19	1.21	0.27	0.86
Maltose	0.13	192	0.98	0.28	0.67	0.75	0.25
Maltose	0.18	318	0.82	0.28	0.93	0.38	0.68
Co-culture:							
Maltose	0.19	7,375[b]	0.04[b]	0.22	1.25	0	0
Maltose	0.24	6,866[b]	0.05[b]	0.40	0.87	0	0
Maltose	0.20	4,069[b]	0.07[b]	0.21	1.37	0	0
Maltose	0.26	8,966[b]	0.04[b]	0.52	0.72	0	0
Monoculture:							
Formate	0.22	-	-	-	-	-	-
Formate	0.29	2.8	149.5	-	-	-	-
Formate	0.11	1.2	132.3	-	-	-	-
Formate	0.10	1.3	111.0	-	-	-	-
Co-culture:							
Formate	0.17	704[b]	0.35[b]	-	-	-	-
Formate	0.17	2,489[b]	0.10[b]	-	-	-	-
Formate	0.24	2,325[b]	0.15[b]	-	-	-	-
Formate	0.11	598[b]	0.27[b]	-	-	-	-
Formate	0.13	1,089[b]	0.17[b]	-	-	-	-

[a]Cell-specific H_2, acetate, or formate production rate = (k/[0.693 × $Y_{product}$])

[b]Based on net H_2 production in co-culture

Table S4. Coding sequences that showed significant and more than two-fold differences in gene expression for high H_2 growth versus low H_2 growth. Expression counts are normalized for size factor.

Coding sequence	Low H_2			High H_2			
	L1	L2	L3	H1	H2	H3	Log_2FC
MJ_RS01685	1370.6	1423.8	2327.9	752.2	447.4	350.9	-1.72
MJ_RS01910	152.9	67.1	210.3	29.7	45.2	32.7	-2.01
MJ_RS03980	2667.1	3121.7	1696.3	858.6	1116.0	804.2	-1.43
MJ_RS09325	326.1	475.5	607.8	240.8	165.0	173.3	-1.28
MJ_RS04390	429.0	447.6	575.4	217.7	79.4	78.1	-1.94
MJ_RS04990	23238.4	23022.4	13108.6	36915.9	55159.0	65542.9	1.41
MJ_RS05550	1059.9	1524.5	1374.3	479.2	465.7	443.3	-1.51
MJ_RS05555	27258.7	35536.4	16861.6	6294.1	8235.9	8493.7	-1.79
MJ_RS06225	1419.6	1450.8	2623.0	846.3	694.3	548.4	-1.39
MJ_RS06835	2919.1	2682.6	3236.1	1016.2	962.0	1140.9	-1.50
MJ_RS06840	2915.3	2684.4	3246.9	1087.1	723.6	910.8	-1.70
MJ_RS06845	1224.4	1766.9	1200.9	352.2	512.1	642.2	-1.48

Table S5. Coding sequences that showed significant and more than two-fold differences in gene expression for co-culture growth versus mono-culture growth. Expression counts are normalized for size factor.

	Co-culture							Mono-culture						
Coding sequence	0.5% Maltose			0.1% formate				High H_2			Low H_2			Log_2 FC
	M1	M2	M3	F1	F2	F3	F4	H1	H2	H3	L1	L2	L3	
MJ_RS00060	415.5	595.8	456.8	473.3	610.1	440.1	300.9	151.3	258.7	268.2	154.7	216.8	236.0	1.12
MJ_RS00100	519.4	1160.2	1636.8	742.9	1261.3	538.9	470.0	411.7	447.4	433.8	394.6	304.1	316.6	1.20
MJ_RS00125	207.7	188.1	342.6	166.8	214.3	154.5	183.2	84.1	100.8	95.3	83.7	75.1	75.3	1.13
MJ_RS00165	519.4	972.1	685.2	388.9	620.6	435.1	450.1	211.0	234.2	237.5	221.8	226.6	238.6	1.23
MJ_RS00180	1558.1	1630.5	1103.9	1513.9	1675.0	1174.2	1274.9	2657.8	2975.0	3780.4	2937.0	2856.9	3954.6	-1.17
MJ_RS00225	51.9	125.4	76.1	99.9	99.2	95.5	85.4	44.8	43.8	28.6	34.5	47.3	46.7	1.20
MJ_RS00230	415.5	282.2	532.9	344.3	296.6	282.3	229.6	101.1	178.0	116.6	106.7	145.1	120.5	1.28
MJ_RS00235	2025.5	2822.1	3273.6	3308.0	3665.7	2788.5	1918.1	359.1	719.9	568.7	401.3	538.7	438.7	2.50
MJ_RS00250	519.4	125.4	152.3	349.1	473.9	316.4	322.4	117.4	190.3	148.4	88.3	159.8	94.5	1.34
MJ_RS00255	207.7	125.4	0.0	128.0	91.8	64.8	62.2	287.8	212.2	251.0	261.6	317.7	313.9	-1.64
MJ_RS00260	103.9	31.4	152.3	60.1	73.9	78.9	75.4	164.7	210.6	256.1	157.5	179.9	129.5	-1.31
MJ_RS00300	0.0	0.0	38.1	26.2	19.0	49.0	41.4	60.6	78.1	99.6	54.9	65.1	66.5	-1.12
MJ_RS00430	0.0	62.7	76.1	195.9	135.1	166.9	155.0	52.9	32.7	28.6	54.3	31.8	106.6	1.41
MJ_RS00435	0.0	62.7	38.1	225.0	157.3	157.8	185.7	46.7	49.3	51.1	48.5	35.6	77.7	1.55
MJ_RS00440	51.9	31.4	0.0	67.9	52.8	73.1	93.7	21.3	23.9	20.4	19.0	14.9	41.4	1.49
MJ_RS00475	259.7	94.1	152.3	183.3	139.3	201.8	203.1	86.5	80.1	60.5	50.5	63.1	78.3	1.36
MJ_RS00490	415.5	689.8	609.1	283.2	311.4	177.7	178.2	945.8	1031.0	1320.8	655.4	886.0	920.5	-1.52
MJ_RS00535	207.7	439.0	342.6	429.6	350.4	448.4	383.0	173.3	178.6	167.4	176.7	198.2	180.0	1.14
MJ_RS00540	831.0	1003.4	1180.0	873.8	813.8	777.3	765.1	207.5	289.5	298.4	260.8	274.4	194.2	1.76
MJ_RS00615	51.9	62.7	76.1	39.8	32.7	47.3	32.3	76.8	59.9	55.7	79.3	84.4	169.8	-1.11
MJ_RS00630	1194.5	878.0	989.7	911.6	942.5	808.8	810.7	316.5	506.9	498.1	326.1	470.0	312.4	1.16
purP	1142.6	1128.8	1294.2	1896.0	1303.5	1518.0	1584.9	5046.5	4464.4	2417.7	3348.8	4669.4	1467.0	-1.32
MJ_RS00710	3168.1	3511.9	3768.5	3630.0	3799.7	2970.4	3349.6	849.4	1397.8	1151.1	782.8	1181.7	568.0	1.81
MJ_RS00720	51.9	0.0	38.1	38.8	38.0	59.0	46.4	82.9	55.2	58.4	114.5	121.4	160.7	-1.18
MJ_RS00730	0.0	31.4	38.1	25.2	24.3	32.4	32.3	5.9	10.1	16.9	5.8	6.4	11.3	1.60
MJ_RS09285	259.7	125.4	342.6	128.0	108.7	162.8	165.0	401.6	420.4	417.5	298.5	387.7	378.2	-1.32
MJ_RS00815	2754454.5	2541703.3	2220143.5	937056.2	1425908.5	949855.6	1049826.0	329786.3	715134.3	895359.2	440040.4	481388.9	518183.6	1.59
MJ_RS00825	278270.7	277725.4	193373.7	102413.2	188071.1	107873.0	121877.8	29600.5	70341.9	81325.0	39350.0	41994.8	56181.0	1.77
MJ_RS00835	779.0	878.0	799.4	1243.3	1168.4	1072.9	1012.1	570.2	558.8	480.5	527.2	536.7	364.1	1.04
MJ_RS00975	51.9	31.4	0.0	22.3	36.9	34.0	31.5	64.7	50.3	47.2	83.5	64.0	64.6	-1.01
MJ_RS01025	103.9	125.4	76.1	103.8	153.0	70.6	92.8	17.0	31.2	29.3	21.3	26.1	28.3	2.04
MJ_RS01050	0.0	62.7	38.1	51.4	54.9	41.5	43.9	101.8	69.8	86.8	102.3	127.3	215.5	-1.32
MJ_RS01090	103.9	31.4	38.1	16.5	19.0	12.5	11.6	44.4	40.2	33.7	43.5	43.9	64.1	-1.37
MJ_RS01110	103.9	313.6	266.5	228.9	240.6	285.7	279.3	515.1	691.1	753.3	509.7	375.4	244.5	-1.02
MJ_RS01135	2025.5	1442.4	1408.4	1345.1	1236.0	1618.5	1364.4	3520.2	4409.8	5107.7	3582.3	3191.8	2505.4	-1.35
MJ_RS01145	831.0	721.2	456.8	464.5	472.9	564.7	518.9	1074.1	1232.5	1720.5	1361.2	955.6	865.6	-1.14
MJ_RS01150	727.1	344.9	304.5	344.3	260.7	414.4	344.0	1113.2	841.1	1183.6	1074.7	899.9	698.8	-1.43
MJ_RS01155	882.9	627.1	304.5	377.3	234.3	463.4	363.1	1339.8	1434.8	1948.8	1286.4	1237.3	1036.4	-1.71
MJ_RS01160	1402.3	658.5	1027.8	1012.5	917.2	1279.7	1064.3	2425.6	1782.5	2377.1	3149.9	1904.3	2116.4	-1.13
MJ_RS01165	415.5	125.4	152.3	139.7	136.2	177.7	169.9	448.0	271.3	281.7	600.8	326.6	346.9	-1.20
MJ_RS01170	259.7	250.9	152.3	164.9	172.0	149.5	141.7	489.7	384.3	586.3	495.2	432.3	878.5	-1.71

MJ_RS01195	0.0	0.0	38.1	29.1	19.0	24.1	9.9	58.0	39.0	45.6	50.1	61.6	96.8	-1.55
MJ_RS01240	0.0	62.7	76.1	57.2	71.8	53.1	59.7	26.5	17.0	13.3	23.8	16.0	25.2	1.55
MJ_RS01275	51.9	156.8	190.3	177.5	176.3	175.2	168.3	75.9	61.3	59.8	78.1	62.0	80.5	1.30
MJ_RS01290	259.7	250.9	228.4	168.7	207.9	266.6	268.6	407.2	604.1	650.9	409.2	419.1	428.3	-1.07
MJ_RS01295	0.0	31.4	76.1	44.6	61.2	48.2	55.5	186.5	152.6	172.5	180.4	168.7	180.8	-1.75
MJ_RS01305	155.8	376.3	342.6	284.2	298.7	216.7	244.5	696.9	528.4	539.4	603.1	597.9	581.5	-1.15
MJ_RS01310	571.3	1003.4	799.4	553.8	661.8	599.6	583.6	1420.3	1470.3	1663.5	1406.0	1394.4	1515.3	-1.21
MJ_RS01320	51.9	250.9	304.5	46.6	70.7	33.2	44.8	15.8	19.1	18.6	17.6	29.3	37.2	1.91
MJ_RS01350	103.9	125.4	114.2	87.3	89.7	85.5	84.5	193.3	162.5	248.0	196.1	181.8	232.2	-1.19
MJ_RS01375	0.0	0.0	76.1	15.5	25.3	30.7	26.5	78.6	76.3	94.8	74.2	73.4	78.7	-1.68
MJ_RS01385	259.7	188.1	114.2	162.9	106.6	160.3	172.4	510.1	304.2	277.8	592.1	499.9	459.8	-1.50
porA	882.9	909.3	456.8	294.8	503.5	587.9	357.3	854.8	945.3	982.6	2230.7	1322.2	807.3	-1.15
MJ_RS01410	51.9	250.9	228.4	66.9	91.8	136.2	82.1	294.4	328.9	268.0	781.8	403.2	242.1	-1.78
MJ_RS01415	207.7	219.5	76.1	51.4	65.4	108.8	51.4	208.0	175.9	160.3	593.0	293.1	151.2	-1.56
MJ_RS01445	0.0	125.4	152.3	104.7	121.4	132.0	125.2	34.6	45.9	30.0	32.5	46.7	56.5	1.53
MJ_RS01455	103.9	344.9	304.5	323.9	310.3	239.2	316.6	644.4	699.0	709.3	505.6	600.0	395.4	-1.02
MJ_RS01480	0.0	62.7	0.0	21.3	25.3	14.9	19.1	52.4	29.6	33.4	49.0	48.1	100.9	-1.35
MJ_RS01510	5609.0	4797.6	3882.7	5525.0	4978.7	4790.6	4615.4	1810.0	2937.3	3269.7	1613.6	2853.2	1646.5	1.05
MJ_RS01515	0.0	31.4	0.0	17.5	6.3	16.6	23.2	31.5	33.1	36.9	30.0	31.6	41.4	-1.09
MJ_RS01535	0.0	0.0	114.2	35.9	35.9	28.2	37.3	112.2	95.8	65.3	63.6	135.7	80.6	-1.42
MJ_RS01545	0.0	62.7	76.1	41.7	59.1	52.3	40.6	103.3	75.1	88.4	228.6	96.3	90.4	-1.22
MJ_RS01560	0.0	0.0	38.1	16.5	16.9	21.6	29.8	58.0	39.0	33.7	44.5	44.7	65.1	-1.18
MJ_RS01570	51.9	31.4	76.1	49.5	41.2	78.1	79.6	161.0	141.6	115.4	134.7	123.3	162.6	-1.18
MJ_RS01610	467.4	1191.6	1218.1	659.5	704.0	423.5	450.1	363.0	221.8	169.0	319.1	419.9	404.4	1.15
MJ_RS01665	103.9	62.7	152.3	38.8	40.1	36.5	31.5	208.8	98.0	111.5	221.0	181.9	366.1	-2.12
MJ_RS01675	2648.7	3041.6	3349.8	2528.3	1229.6	2440.6	1637.9	6560.7	8474.3	6726.0	5228.8	5455.6	2590.8	-1.30
MJ_RS01680	779.0	439.0	609.1	749.7	728.3	1118.6	1340.3	340.2	392.0	399.7	318.4	401.6	397.4	1.21
MJ_RS01705	0.0	94.1	76.1	61.1	57.0	54.0	49.7	100.8	76.9	85.2	115.7	103.5	211.8	-1.04
MJ_RS01720	0.0	219.5	114.2	7.8	9.5	14.9	15.7	4.0	6.1	6.4	4.2	7.5	19.6	2.38
MJ_RS01755	0.0	0.0	38.1	9.7	7.4	6.6	9.1	9.7	24.8	38.5	17.9	18.1	27.6	-1.40
MJ_RS01845	0.0	0.0	38.1	9.7	5.3	14.1	7.5	19.4	21.1	19.0	29.7	22.1	55.1	-1.54
MJ_RS01865	103.9	282.2	0.0	67.9	87.6	53.1	30.7	277.1	174.5	198.3	270.0	279.7	402.4	-1.89
MJ_RS01870	51.9	156.8	114.2	43.6	66.5	33.2	25.7	138.2	63.5	69.2	148.5	140.2	233.7	-1.31
MJ_RS01900	363.5	689.8	647.1	614.9	562.6	759.0	673.9	225.3	189.3	131.7	225.8	309.8	303.3	1.45
MJ_RS01910	0.0	0.0	0.0	1.0	2.1	0.8	5.8	4.9	7.3	5.3	24.7	11.1	36.4	-2.26
MJ_RS01915	103.9	62.7	0.0	25.2	39.1	31.6	36.5	66.1	66.4	90.2	171.1	102.9	263.6	-1.85
MJ_RS01965	934.8	878.0	456.8	803.0	966.8	728.3	709.5	2071.6	2580.8	2989.6	2463.1	1852.7	2265.8	-1.59
MJ_RS01995	51.9	31.4	38.1	27.2	27.4	26.6	25.7	70.3	79.3	86.1	56.9	67.6	78.2	-1.43
MJ_RS02005	311.6	188.1	342.6	220.1	174.2	246.6	202.3	458.4	619.9	646.1	544.3	398.1	565.6	-1.29
MJ_RS02010	155.8	94.1	190.3	81.5	49.6	78.9	95.3	243.9	284.3	313.3	342.0	193.1	301.2	-1.73
MJ_RS02015	103.9	125.4	76.1	101.8	95.0	142.0	112.7	430.6	502.8	486.7	545.7	338.2	453.8	-2.03
MJ_RS02020	207.7	250.9	418.7	194.0	206.9	146.2	135.1	514.7	433.6	442.0	479.8	458.8	471.5	-1.31
MJ_RS02025	1298.4	2069.5	2398.1	1461.5	1716.2	1738.1	1995.2	785.1	1018.4	917.3	752.4	1002.3	884.0	1.01
MJ_RS02030	1246.5	1975.5	2207.8	1605.0	1712.0	2254.6	2440.3	311.3	584.6	527.5	321.2	568.5	378.8	2.11
MJ_RS02100	1194.5	2069.5	2436.2	702.1	775.8	769.8	817.3	237.4	69.4	88.4	211.2	271.3	661.1	2.26
MJ_RS02115	623.2	344.9	799.4	750.6	527.7	734.1	622.5	2133.0	1943.1	2324.0	2203.0	1716.8	1643.7	-1.64
MJ_RS02130	1765.8	1317.0	1827.2	1617.6	1399.6	1088.7	1092.5	646.0	607.3	497.7	560.4	875.3	637.1	1.14
MJ_RS02285	0.0	31.4	0.0	20.4	20.1	18.3	25.7	53.0	43.0	43.7	42.1	57.7	72.3	-1.33
MJ_RS02405	0.0	0.0	38.1	9.7	11.6	7.5	13.3	31.0	20.7	19.9	24.9	21.5	47.8	-1.37
MJ_RS02415	103.9	188.1	228.4	148.4	133.0	134.5	127.7	358.9	228.3	235.2	289.7	276.7	308.3	-1.02

MJ_RS02420	675.2	313.6	342.6	375.3	443.3	432.6	466.7	916.1	947.6	1207.9	891.6	797.4	661.3	-1.08
MJ_RS02440	103.9	219.5	152.3	264.8	323.0	222.6	274.4	460.3	513.4	645.6	747.5	491.8	600.4	-1.19
rps14P	259.7	250.9	228.4	220.1	183.7	124.6	135.9	249.0	363.8	484.6	442.5	259.9	370.5	-1.01
MJ_RS02550	727.1	815.3	685.2	517.9	608.0	709.2	702.1	1400.3	2038.8	2293.3	1300.5	1499.9	936.3	-1.24
MJ_RS02640	51.9	376.3	266.5	226.9	283.9	230.9	180.7	544.5	532.0	597.3	394.4	497.3	531.4	-1.15
MJ_RS02705	1713.9	2383.1	2474.3	4606.6	3525.3	3731.0	4039.3	1067.0	1557.7	1654.3	1255.0	1040.6	1068.3	1.37
MJ_RS02710	467.4	595.8	1142.0	1762.1	1165.2	1190.0	1250.8	487.2	606.5	598.9	670.2	450.3	526.6	1.03
MJ_RS02730	207.7	250.9	228.4	141.6	383.1	230.9	205.6	337.3	1115.2	862.8	280.9	455.4	182.0	-1.18
MJ_RS02745	0.0	31.4	76.1	50.4	126.7	116.3	102.0	121.6	403.2	442.0	122.2	161.7	70.6	-1.37
MJ_RS02750	51.9	94.1	266.5	88.3	183.7	181.9	164.1	339.2	1545.8	1344.9	333.6	460.6	177.5	-2.22
MJ_RS02755	311.6	439.0	304.5	179.4	408.5	382.0	324.9	886.2	2472.6	2468.1	778.3	1051.2	444.9	-2.02
MJ_RS02760	103.9	94.1	76.1	63.0	121.4	129.5	103.6	216.5	588.8	597.8	216.5	269.9	113.7	-1.72
MJ_RS02765	207.7	219.5	304.5	98.9	207.9	196.8	181.5	493.1	1282.2	1190.7	625.7	634.2	294.4	-1.99
MJ_RS02770	519.4	94.1	190.3	116.4	212.2	210.1	186.5	426.8	918.6	927.4	549.0	478.7	231.5	-1.57
MJ_RS02775	259.7	250.9	418.7	161.0	320.9	311.4	263.6	736.6	2126.9	2285.5	1108.0	919.5	431.6	-2.19
MJ_RS02780	51.9	156.8	152.3	106.7	175.2	157.8	179.9	412.0	1034.4	1015.1	654.3	479.6	200.9	-2.09
MJ_RS02785	51.9	219.5	76.1	47.5	122.4	115.4	118.5	383.4	791.5	747.6	573.3	418.4	176.0	-2.28
MJ_RS02790	0.0	219.5	152.3	45.6	83.4	89.7	81.2	248.5	557.8	486.9	414.6	311.5	134.0	-2.06
MJ_RS02795	363.5	470.3	266.5	97.0	268.1	245.8	259.4	599.9	1131.4	873.5	1105.8	714.2	263.2	-1.58
MJ_RS02800	363.5	282.2	342.6	94.1	297.6	218.4	208.9	568.4	967.9	657.3	992.6	668.8	224.5	-1.51
MJ_RS02805	779.0	313.6	342.6	135.8	339.9	231.7	290.1	1365.6	1837.3	1253.0	1990.1	1468.4	448.8	-2.15
MJ_RS02850	207.7	439.0	190.3	464.5	486.6	469.2	518.1	258.4	161.9	112.5	242.0	206.5	220.3	1.11
MJ_RS02860	51.9	62.7	0.0	100.9	112.9	131.2	126.0	46.4	44.8	30.0	45.7	46.1	51.8	1.28
MJ_RS02865	0.0	250.9	266.5	226.9	258.6	275.7	304.2	113.4	107.7	79.5	110.1	94.9	110.0	1.31
MJ_RS02940	259.7	94.1	228.4	166.8	109.8	168.6	126.8	382.1	490.4	490.4	350.4	323.5	254.2	-1.36
MJ_RS02945	51.9	94.1	76.1	98.0	68.6	101.3	67.1	227.7	219.8	190.1	205.5	177.0	149.5	-1.23
MJ_RS02995	0.0	250.9	76.1	68.9	61.2	73.9	115.2	331.1	242.9	290.6	194.9	52.2	121.8	-1.21
MJ_RS03005	51.9	125.4	38.1	44.6	36.9	40.7	38.1	82.6	81.4	89.3	79.0	71.5	143.7	-1.09
MJ_RS03015	0.0	31.4	0.0	6.8	11.6	6.6	16.6	22.9	18.3	24.7	20.4	18.7	23.8	-1.02
MJ_RS03035	51.9	62.7	38.1	43.6	42.2	46.5	53.1	88.5	71.8	92.1	106.2	99.9	139.1	-1.09
MJ_RS03055	51.9	0.0	0.0	27.2	17.9	26.6	24.9	39.1	38.6	41.9	61.6	39.3	86.7	-1.12
MJ_RS03060	259.7	282.2	228.4	161.0	136.2	170.2	222.1	342.5	439.3	499.8	340.8	383.1	295.7	-1.05
MJ_RS03075	155.8	125.4	304.5	422.8	160.4	176.0	148.4	572.2	641.8	493.6	379.8	456.8	393.6	-1.16
MJ_RS03095	207.7	0.0	266.5	68.9	83.4	68.1	63.0	414.8	494.7	547.4	385.1	411.3	611.4	-2.58
MJ_RS03100	311.6	156.8	380.7	85.3	111.9	93.8	88.7	917.1	1045.4	1182.5	722.0	734.5	939.8	-2.82
MJ_RS03105	1713.9	1881.4	1636.8	1960.0	1946.3	1057.1	1088.4	723.5	890.6	635.6	612.5	850.4	750.6	1.10
MJ_RS03260	51.9	31.4	114.2	63.0	48.6	62.3	41.4	151.7	92.5	116.6	170.8	128.4	95.2	-1.20
MJ_RS03285	259.7	282.2	190.3	331.7	343.0	284.0	335.7	165.6	132.9	88.9	156.9	181.9	165.2	1.05
MJ_RS03310	467.4	595.8	609.1	576.1	480.2	700.9	690.5	259.6	259.5	165.4	229.6	249.5	209.8	1.39
MJ_RS03330	103.9	188.1	76.1	162.9	203.7	255.8	315.8	79.9	61.5	46.7	61.7	85.2	90.4	1.58
MJ_RS03335	103.9	62.7	152.3	217.2	207.9	246.6	272.7	90.3	67.8	57.7	61.6	90.6	110.4	1.40
MJ_RS03380	51.9	0.0	38.1	48.5	68.6	70.6	75.4	24.2	17.5	17.4	25.2	22.3	33.9	1.40
MJ_RS03395	0.0	0.0	0.0	48.5	47.5	39.0	34.8	87.1	96.4	96.9	63.8	81.5	70.7	-1.11
MJ_RS03420	0.0	31.4	0.0	32.0	43.3	29.9	34.8	52.5	52.4	54.3	57.7	69.7	136.5	-1.10
MJ_RS09320	0.0	156.8	114.2	74.7	46.4	72.2	92.0	201.3	216.1	171.5	128.0	197.4	203.8	-1.33
MJ_RS03470	51.9	407.6	380.7	265.7	353.6	227.5	201.4	85.4	136.2	112.7	78.2	128.5	103.3	1.33
MJ_RS03480	13191.6	11570.6	11001.0	15261.0	10141.0	14246.5	18949.7	439.9	881.4	1033.4	423.7	584.5	249.4	4.49
MJ_RS03490	51.9	156.8	0.0	36.9	34.8	34.0	49.7	114.3	82.8	105.6	97.8	100.1	146.1	-1.38
MJ_RS03495	623.2	815.3	1065.8	647.8	541.5	631.9	639.1	1461.9	2071.3	1734.9	1076.7	1965.7	1045.0	-1.20

MJ_RS03510	103.9	188.1	190.3	79.5	69.7	59.8	65.5	240.1	125.4	150.7	243.6	252.7	376.3	-1.46
MJ_RS03515	1817.7	2289.0	2854.9	3071.4	2653.5	2370.0	2354.1	889.9	1196.8	1114.7	887.9	1190.2	731.3	1.33
MJ_RS03525	103.9	501.7	418.7	205.6	169.9	105.5	127.7	97.9	55.0	49.5	72.8	101.0	182.4	1.18
MJ_RS03540	103.9	94.1	76.1	32.0	43.3	48.2	52.2	94.5	59.7	90.0	94.4	92.1	149.1	-1.00
MJ_RS03545	155.8	188.1	266.5	251.2	235.4	166.9	175.7	452.6	276.2	320.6	551.0	415.5	471.4	-1.00
MJ_RS03570	51.9	94.1	38.1	80.5	83.4	72.2	66.3	147.0	128.2	135.4	139.5	154.3	205.8	-1.02
MJ_RS03645	0.0	62.7	76.1	79.5	114.0	31.6	38.1	13.5	22.1	23.1	15.2	14.6	19.0	1.83
MJ_RS03650	0.0	62.7	0.0	42.7	47.5	32.4	39.0	93.4	73.3	82.9	86.9	96.3	145.3	-1.29
MJ_RS03685	51.9	0.0	0.0	27.2	31.7	27.4	19.1	77.2	50.5	53.8	82.6	67.1	76.9	-1.41
MJ_RS03710	103.9	31.4	76.1	52.4	77.0	63.1	78.7	180.2	122.2	120.5	124.0	124.8	188.4	-1.08
MJ_RS03720	259.7	188.1	152.3	72.7	106.6	83.9	118.5	272.1	214.1	192.6	219.6	245.0	291.7	-1.14
MJ_RS03725	415.5	344.9	342.6	154.2	203.7	186.8	197.3	772.1	715.0	818.1	575.5	724.7	523.4	-1.66
MJ_RS03730	311.6	125.4	190.3	68.9	109.8	73.1	105.3	386.6	251.6	262.2	432.2	496.4	789.0	-1.98
MJ_RS03735	0.0	0.0	0.0	36.9	54.9	28.2	36.5	82.2	76.7	93.4	75.6	87.4	113.2	-1.32
MJ_RS03775	980439.5	978075.1	811522.5	387782.5	661575.8	382269.2	434893.1	108314.5	295432.6	336238.7	146626.8	164120.4	195537.2	1.67
MJ_RS03785	3529800.5	3266824.6	2921960.7	1156300.7	1820402.3	1141648.3	1273683.8	396594.1	902399.9	1106603.1	522575.5	593058.1	646991.9	1.64
MJ_RS03810	51.9	156.8	304.5	108.6	83.4	98.8	110.2	207.7	264.8	259.7	210.6	236.7	231.1	-1.12
MJ_RS03815	103.9	0.0	0.0	20.4	15.8	34.0	33.2	62.2	85.6	88.2	36.2	53.1	45.8	-1.25
MJ_RS03820	0.0	31.4	0.0	37.8	46.4	43.2	32.3	125.4	93.9	78.1	49.9	52.0	63.8	-1.05
MJ_RS03825	155.8	125.4	76.1	76.6	83.4	103.0	60.5	176.2	238.0	253.1	151.2	182.1	184.8	-1.23
MJ_RS03845	207.7	156.8	190.3	196.9	214.3	165.3	152.5	479.1	507.3	544.0	427.9	279.9	276.3	-1.20
MJ_RS03890	103.9	721.2	532.9	381.1	1802.8	497.4	393.7	228.8	275.3	238.4	286.6	167.8	149.1	1.55
MJ_RS03895	727.1	1693.3	1218.1	1126.9	5720.7	1694.9	1314.7	531.4	712.4	647.2	830.0	340.1	307.0	1.80
MJ_RS03900	155.8	439.0	228.4	307.4	1154.7	351.3	359.7	145.6	132.9	100.8	226.6	89.5	46.2	1.86
MJ_RS03910	2804.5	4045.0	2854.9	3284.8	10399.6	3766.7	2548.1	1706.6	2176.8	1605.5	3552.6	1447.7	505.3	1.22
MJ_RS03915	5349.4	8372.2	6433.1	6796.4	23239.5	8091.5	5630.0	3236.7	4312.4	3248.8	7065.5	3043.2	722.0	1.34
MJ_RS03920	4881.9	8873.9	6433.1	4497.0	17620.1	5660.9	4132.1	2460.9	3976.6	3361.1	6095.6	2523.5	501.1	1.24
MJ_RS03950	4985.8	6522.2	6471.2	5698.6	9484.5	4297.4	4019.4	2185.6	2837.7	2125.0	3926.0	2984.0	961.7	1.24
MJ_RS03980	934.8	1379.7	1065.8	723.5	1767.9	675.1	733.6	140.5	180.0	129.6	431.3	514.0	293.9	1.88
MJ_RS04015	155.8	815.3	342.6	199.8	235.4	284.0	280.2	70.3	101.9	107.4	75.1	112.7	88.6	1.71
MJ_RS04020	103.9	282.2	380.7	120.3	164.7	181.0	164.1	40.9	52.1	46.5	40.0	55.0	45.1	1.89
MJ_RS04030	259.7	250.9	342.6	107.6	91.8	93.0	102.8	51.3	40.2	32.8	24.3	61.7	39.5	1.74
MJ_RS04035	415.5	783.9	989.7	419.0	313.5	292.3	299.2	205.3	217.1	215.7	107.8	270.8	190.4	1.19
MJ_RS04070	727.1	1160.2	1408.4	885.4	740.9	799.7	848.0	276.3	426.7	403.6	297.4	454.1	300.0	1.33
MJ_RS04075	934.8	1254.3	685.2	837.9	641.7	685.9	588.5	201.4	247.5	224.0	194.9	319.8	181.2	1.75
MJ_RS04080	7219.0	9124.8	9706.8	12003.4	9576.3	8700.2	8892.5	2926.2	3845.1	3139.6	2965.1	4626.7	2193.2	1.51
MJ_RS04140	727.1	1034.8	532.9	614.9	620.6	610.4	684.7	239.9	368.5	224.5	217.3	390.9	321.8	1.19
MJ_RS04145	363.5	501.7	304.5	425.7	405.3	430.2	567.0	132.4	115.5	87.0	117.4	164.9	155.1	1.79
MJ_RS04175	0.0	94.1	38.1	45.6	55.9	44.8	45.6	101.5	81.6	94.4	79.6	102.1	140.9	-1.06
MJ_RS04180	4881.9	4797.6	4986.6	9404.3	4589.2	6483.9	4760.4	14656.0	13331.3	11160.1	9560.6	14141.5	10680.1	-1.09
MJ_RS04205	51.9	31.4	38.1	69.8	74.9	88.9	111.1	25.2	54.2	35.5	29.9	31.0	34.6	1.20
MJ_RS04230	51.9	94.1	0.0	13.6	20.1	19.9	18.2	61.1	31.5	31.6	41.1	70.9	89.8	-1.40
MJ_RS04255	51.9	31.4	38.1	19.4	21.1	21.6	19.1	46.6	37.7	38.9	35.5	47.7	54.7	-1.05
MJ_RS04260	0.0	31.4	0.0	1.9	7.4	5.0	9.1	34.0	19.3	23.4	26.3	30.2	38.8	-2.17
MJ_RS04290	519.4	595.8	1142.0	614.9	530.9	694.2	804.9	328.5	402.2	333.0	271.7	357.4	204.3	1.12
MJ_RS04330	103.9	31.4	114.2	52.4	68.6	45.7	68.8	119.6	149.3	134.4	107.5	154.6	89.1	-1.06
MJ_RS04380	51.9	62.7	114.2	96.0	92.9	91.3	88.7	199.8	183.4	216.9	150.7	197.4	223.1	-1.10

MJ_RS09325	51.9	62.7	114.2	202.7	255.4	195.1	150.9	39.4	26.6	27.9	52.7	78.3	105.3	1.62
MJ_RS04390	155.8	156.8	304.5	477.1	487.6	399.4	290.9	35.6	12.8	12.6	69.4	73.7	99.7	2.75
MJ_RS09330	259.7	282.2	190.3	547.9	496.1	364.6	279.3	39.8	6.7	7.3	92.5	65.1	69.9	2.92
MJ_RS04395	162558.3	155999.1	155840.9	228882.6	165805.8	177880.8	175952.6	79914.0	91522.2	104158.4	103433.0	88903.7	50627.1	1.02
MJ_RS04410	103.9	125.4	152.3	484.9	345.1	245.8	233.8	1280.3	1194.5	865.1	846.7	964.3	712.3	-1.81
MJ_RS04460	207.7	188.1	380.7	535.3	563.6	577.1	641.6	231.4	259.5	198.8	202.5	273.7	264.8	1.06
MJ_RS04485	727.1	1473.8	1408.4	345.3	505.6	214.2	245.4	242.7	240.2	267.1	186.3	243.3	290.8	1.43
MJ_RS04500	415.5	439.0	456.8	315.2	491.9	399.4	424.4	205.3	65.5	74.0	147.1	193.1	347.9	1.28
MJ_RS04625	3531.6	2100.9	1522.6	2037.6	1068.1	2156.6	1864.2	9894.2	9868.2	7923.4	7198.4	7713.3	4057.1	-1.96
MJ_RS04630	1506.1	1034.8	1370.4	1061.9	516.1	1148.5	1038.6	5747.4	5634.4	4020.7	3889.7	4361.5	2327.2	-2.03
MJ_RS04660	882.9	627.1	571.0	219.2	210.0	164.4	152.5	585.4	707.3	715.3	1161.9	737.0	770.7	-1.16
MJ_RS04860	51.9	31.4	38.1	52.4	78.1	88.0	81.2	40.1	38.6	31.6	28.0	33.5	31.2	1.09
MJ_RS04940	259.7	313.6	380.7	257.0	255.4	372.9	335.7	985.4	1257.6	1338.0	1087.8	904.9	434.0	-1.70
MJ_RS04965	467.4	1222.9	1370.4	1389.7	1361.6	1285.5	1322.1	287.5	354.1	265.7	304.2	371.7	326.0	1.99
MJ_RS04970	571.3	1379.7	1218.1	1263.7	1432.3	1426.6	1183.7	380.8	409.9	399.9	285.4	447.2	294.7	1.76
MJ_RS04990	3479.7	2445.8	2055.5	1772.8	2782.2	2688.0	2318.5	6041.4	8897.9	10565.3	3757.7	3790.9	2271.0	-1.24
MJ_RS04995	0.0	0.0	0.0	9.7	5.3	3.3	5.0	10.8	27.4	32.1	13.8	16.7	16.1	-1.71
MJ_RS05035	259.7	407.6	494.9	387.0	608.0	394.4	346.5	182.4	59.5	59.5	211.8	204.5	303.2	1.30
MJ_RS05040	103.9	125.4	152.3	171.7	225.9	212.6	225.5	51.6	23.1	19.2	71.7	65.3	127.6	1.64
MJ_RS05080	51.9	188.1	152.3	144.5	80.2	88.9	67.1	19.2	24.3	23.6	29.7	24.7	31.1	1.98
MJ_RS05085	467.4	344.9	152.3	217.2	288.1	330.5	279.3	523.6	628.8	570.1	450.7	729.3	528.2	-1.01
MJ_RS05135	519.4	282.2	152.3	406.4	391.6	385.3	366.4	715.9	988.4	1026.3	406.7	887.3	648.7	-1.08
MJ_RS05165	51.9	188.1	114.2	104.7	100.3	103.0	103.6	234.9	190.7	218.0	198.9	207.7	231.4	-1.04
MJ_RS05170	467.4	439.0	609.1	406.4	277.6	460.0	374.7	1312.7	1037.5	1297.9	1125.1	957.1	857.9	-1.44
MJ_RS05175	571.3	564.4	571.0	617.8	657.6	590.4	564.5	1285.6	1662.0	1828.4	774.4	988.6	731.3	-1.02
MJ_RS05225	103.9	94.1	190.3	253.1	63.3	176.9	132.6	466.6	301.1	275.1	373.1	308.9	271.3	-1.14
MJ_RS05325	0.0	94.1	114.2	38.8	42.2	44.0	40.6	89.5	102.9	123.4	99.4	109.8	151.2	-1.37
MJ_RS05365	363.5	501.7	723.2	676.9	451.7	485.0	756.0	406.6	270.3	231.8	250.9	225.1	194.6	1.13
MJ_RS05375	51.9	94.1	38.1	38.8	40.1	36.5	35.6	79.5	88.3	101.5	87.6	61.6	84.9	-1.10
MJ_RS05415	363.5	344.9	342.6	334.6	219.5	326.4	234.6	671.9	633.5	745.0	698.0	647.4	469.4	-1.15
MJ_RS05455	2025.5	1912.8	1560.7	4053.8	1942.1	3820.7	4034.3	1438.0	1759.2	2296.7	932.2	1251.9	689.8	1.01
MJ_RS05470	207.7	250.9	228.4	208.5	176.3	181.0	177.4	402.1	326.1	414.3	414.5	353.9	379.6	-1.01
MJ_RS05490	779.0	1034.8	456.8	659.5	632.2	662.7	621.7	1427.7	1389.5	1545.3	1369.3	1401.3	1755.6	-1.15
MJ_RS05495	0.0	62.7	0.0	9.7	13.7	11.6	10.8	46.3	33.3	28.6	39.2	42.5	64.3	-1.82
MJ_RS05520	727.1	407.6	571.0	862.2	689.2	724.9	891.9	372.4	229.9	202.5	424.4	400.6	388.2	1.10
MJ_RS05525	103.9	94.1	38.1	232.8	190.0	239.2	273.5	79.2	55.2	41.9	71.5	80.0	90.0	1.52
MJ_RS05530	207.7	62.7	152.3	86.3	78.1	98.8	110.2	38.7	25.4	22.9	30.6	46.1	69.7	1.35
MJ_RS05550	467.4	940.7	837.4	325.9	350.4	238.3	223.0	78.4	75.1	71.5	171.4	251.0	238.1	1.62
MJ_RS05555	13295.5	14392.7	14807.6	9795.1	11398.1	10263.1	11114.8	1030.1	1328.6	1369.2	4407.8	5851.4	2921.2	2.11
MJ_RS09345	0.0	0.0	38.1	11.6	17.9	18.3	14.9	53.9	53.2	54.3	45.9	54.4	73.7	-1.84
MJ_RS05720	207.7	125.4	228.4	105.7	140.4	142.8	144.2	305.5	281.0	286.5	247.3	363.9	380.2	-1.17
MJ_RS05870	311.6	501.7	532.9	385.0	395.8	427.7	460.9	818.1	972.7	1152.7	883.2	839.4	684.4	-1.07
MJ_RS05885	103.9	125.4	114.2	135.8	103.4	172.7	126.0	468.1	404.6	556.8	461.9	338.8	299.5	-1.67
MJ_RS05900	779.0	972.1	875.5	627.5	724.1	445.9	416.9	325.0	372.3	311.9	255.1	394.0	268.1	1.01
MJ_RS05930	103.9	376.3	114.2	170.7	135.1	176.0	157.5	720.3	524.5	579.7	691.5	552.9	585.1	-1.88
MJ_RS05940	415.5	752.6	951.6	991.1	907.7	976.6	1090.8	342.5	267.2	245.1	316.3	317.4	327.1	1.61
MJ_RS05975	155.8	188.1	114.2	168.7	191.0	121.2	158.3	436.8	359.1	335.8	369.7	330.2	302.0	-1.16
MJ_RS05995	103.9	31.4	38.1	33.0	17.9	38.2	40.6	125.1	108.1	124.1	98.0	103.2	94.2	-1.69

MJ_RS06000	51.9	0.0	0.0	22.3	8.4	26.6	22.4	56.3	51.9	69.4	54.4	49.9	49.5	-1.47
MJ_RS06110	207.7	188.1	418.7	268.6	194.2	191.8	195.6	86.7	73.9	45.8	66.7	115.6	124.9	1.39
MJ_RS06125	51.9	156.8	38.1	143.5	157.3	126.2	112.7	295.1	240.4	301.6	281.2	261.2	319.4	-1.12
MJ_RS06135	311.6	62.7	76.1	105.7	134.0	144.5	160.8	346.9	306.8	419.4	361.1	286.6	316.8	-1.32
MJ_RS06170	103.9	0.0	38.1	52.4	79.2	44.8	48.1	22.0	13.8	9.2	18.5	24.9	36.0	1.38
MJ_RS06180	9504.2	12103.6	10886.8	11244.0	10067.1	8592.3	8987.0	3958.4	6065.1	5130.6	4564.1	4539.5	2796.1	1.18
MJ_RS06185	779.0	721.2	532.9	644.9	559.4	614.5	557.9	212.2	350.4	240.0	263.3	297.9	198.6	1.23
MJ_RS06205	3375.8	3386.5	2360.1	1445.0	2076.1	2122.5	2236.4	464.9	290.8	346.3	790.1	674.5	787.4	2.10
MJ_RS06210	415.5	313.6	266.5	239.5	308.2	338.8	257.0	134.7	78.7	68.3	151.0	197.3	257.2	1.00
MJ_RS06230	2856.5	2038.2	2245.9	2284.9	1955.8	2186.5	2130.3	5651.5	6118.5	6142.4	3740.0	4562.0	1669.6	-1.06
MJ_RS06265	571.3	156.8	76.1	187.2	218.5	300.6	308.4	514.3	709.4	765.7	462.2	661.1	413.6	-1.22
MJ_RS06325	882.9	1285.6	989.7	1004.7	955.2	897.7	853.8	298.0	378.8	377.7	323.8	420.8	410.0	1.38
MJ_RS06330	311.6	31.4	152.3	112.5	96.0	89.7	64.7	714.2	579.7	643.1	616.0	663.6	471.9	-2.67
MJ_RS06355	9452.3	7494.2	7575.1	7591.7	7293.3	7600.8	7451.9	13062.4	26008.8	29556.8	12393.4	14148.1	8422.2	-1.15
MJ_RS06390	51.9	62.7	0.0	17.5	32.7	20.8	24.9	78.3	51.9	60.9	92.7	97.6	236.8	-2.03
MJ_RS06395	467.4	344.9	418.7	361.7	312.4	318.0	326.6	676.5	527.6	656.2	807.7	530.1	1068.5	-1.05
MJ_RS06445	0.0	470.3	304.5	44.6	32.7	8.3	16.6	3.2	5.5	4.1	5.3	4.1	7.3	4.52
MJ_RS06450	155.8	1630.5	1751.0	450.0	432.7	83.0	104.4	30.1	66.1	71.5	36.1	30.9	35.0	3.84
MJ_RS06455	675.2	972.1	1522.6	414.1	785.3	270.7	321.6	206.4	17.4	14.4	103.9	105.2	114.0	2.90
MJ_RS06520	0.0	0.0	0.0	3.9	2.1	6.6	4.1	14.8	13.6	18.1	16.6	18.6	27.1	-2.00
MJ_RS06525	0.0	62.7	114.2	27.2	15.8	31.6	36.5	115.3	103.5	110.4	94.2	103.3	142.0	-1.89
MJ_RS06550	0.0	0.0	76.1	47.5	28.5	49.8	47.2	90.6	73.7	90.9	88.8	76.8	122.9	-1.10
MJ_RS06575	727.1	1034.8	1370.4	1096.9	1481.9	1003.1	1118.2	556.9	699.2	656.6	478.1	570.2	314.4	1.06
MJ_RS06580	51.9	62.7	76.1	69.8	119.3	85.5	96.2	51.0	53.0	44.7	38.1	47.9	27.1	1.04
MJ_RS06605	1298.4	1191.6	1408.4	1298.6	1300.3	1042.2	1097.5	461.9	755.4	704.3	460.4	546.9	454.3	1.11
MJ_RS06640	363.5	501.7	380.7	353.0	288.1	307.3	333.2	1060.8	832.5	711.4	1123.1	885.1	856.1	-1.44
MJ_RS06650	155.8	156.8	152.3	130.9	141.4	166.9	150.0	329.5	344.1	368.7	408.6	306.9	242.6	-1.17
MJ_RS06665	363.5	501.7	380.7	141.6	116.1	70.6	91.2	39.5	9.1	9.8	30.9	33.5	27.2	3.03
MJ_RS06670	51.9	282.2	190.3	94.1	116.1	110.4	72.9	292.0	365.8	450.7	298.8	201.7	307.6	-1.52
MJ_RS06675	0.0	31.4	152.3	15.5	27.4	28.2	17.4	95.0	104.7	93.2	82.0	80.0	254.9	-2.17
MJ_RS06685	207.7	125.4	38.1	46.6	46.4	49.8	35.6	121.5	74.5	97.8	97.2	104.9	143.2	-1.01
MJ_RS06840	882.9	940.7	571.0	713.8	612.2	633.6	689.7	177.9	116.7	146.8	471.4	442.0	562.5	1.15
MJ_RS06845	779.0	940.7	799.4	844.7	565.7	875.3	965.7	57.6	82.6	103.5	198.0	290.9	208.1	2.39
MJ_RS06850	17086.8	15960.5	15683.1	11763.8	9877.1	12633.9	13483.9	735.3	1113.6	1281.0	2155.6	2642.0	1627.8	3.11
MJ_RS09360	415.5	689.8	456.8	400.5	387.4	634.4	718.7	149.7	190.1	163.8	144.0	250.4	196.6	1.55
MJ_RS06855	1454.2	1818.7	1560.7	1395.6	1457.6	1515.5	1818.6	261.7	321.0	381.3	410.7	571.1	415.6	2.00
MJ_RS06860	207.7	376.3	456.8	354.0	312.4	282.3	347.3	123.1	135.5	149.1	135.8	188.8	198.6	1.09
MJ_RS06995	51.9	31.4	38.1	37.8	51.7	61.5	42.3	117.2	140.4	140.9	242.5	129.3	118.1	-1.63
MJ_RS07000	51.9	219.5	76.1	92.1	104.5	91.3	62.2	157.4	160.1	147.5	578.1	192.2	205.9	-1.35
MJ_RS07045	311.6	313.6	494.9	450.0	561.5	394.4	404.5	180.5	199.9	235.7	202.8	204.0	222.5	1.08
MJ_RS07180	51.9	0.0	0.0	37.8	29.6	29.9	24.9	70.6	77.1	100.8	74.8	83.5	84.5	-1.46
MJ_RS07190	103.9	94.1	114.2	174.6	149.9	202.6	155.0	469.3	592.5	550.6	324.4	442.3	334.8	-1.49
MJ_RS07195	2129.4	1787.3	1598.8	2075.4	1181.1	1787.0	1356.1	7276.0	6673.5	7454.4	4105.1	4356.9	2739.1	-1.69
MJ_RS07230	103.9	188.1	114.2	122.2	122.4	112.1	97.0	236.8	251.2	342.9	233.8	238.1	303.7	-1.21
MJ_RS07245	0.0	0.0	0.0	2.9	3.2	5.8	3.3	22.5	18.1	21.5	16.5	21.3	39.5	-2.49
MJ_RS07270	1402.3	1505.1	1332.3	1626.4	1319.3	1655.8	1553.4	554.2	916.1	790.6	564.4	631.2	392.8	1.22
MJ_RS07275	207.7	533.1	266.5	563.5	456.0	513.2	485.7	214.4	233.7	161.7	173.9	238.4	143.2	1.27
MJ_RS07310	207.7	407.6	456.8	277.4	239.6	244.1	265.3	641.2	533.2	642.4	698.8	527.7	773.9	-1.22

MJ_RS07315	103.9	62.7	114.2	33.0	31.7	39.0	31.5	162.0	83.8	87.9	145.0	113.9	221.1	-1.77
MJ_RS07430	0.0	31.4	38.1	57.2	73.9	94.7	87.0	23.8	16.2	10.8	23.6	25.5	28.0	1.73
MJ_RS07495	51.9	0.0	0.0	65.0	50.7	97.2	89.5	95.8	161.5	165.1	97.8	112.4	249.4	-1.15
MJ_RS07595	571.3	439.0	494.9	324.9	335.6	201.8	194.8	787.4	567.9	309.2	691.6	935.8	739.5	-1.04
MJ_RS07715	4518.4	3543.3	4073.0	1091.0	782.1	1638.4	1821.1	332.9	687.7	868.0	608.3	907.2	597.0	1.87
MJ_RS07720	3220.0	2383.1	2626.5	1120.1	1088.2	1795.4	2003.5	275.0	393.0	454.2	400.5	563.0	490.7	2.20
MJ_RS07750	103.9	62.7	152.3	185.2	152.0	176.9	219.7	81.8	88.7	72.1	64.7	72.8	66.6	1.22
MJ_RS07770	207.7	439.0	685.2	322.0	368.4	391.1	379.6	185.6	143.7	123.2	145.0	185.5	148.8	1.30
MJ_RS07775	207.7	156.8	532.9	391.8	371.5	343.8	329.1	211.4	140.6	109.7	137.8	186.7	177.8	1.11
MJ_RS07780	4206.8	4578.1	6090.5	5366.9	5714.3	4046.6	4079.9	1246.0	1422.2	951.4	910.3	995.3	773.1	2.21
MJ_RS07785	155.8	156.8	304.5	139.7	149.9	142.0	103.6	350.7	248.8	320.0	318.7	229.7	258.0	-1.02
MJ_RS07835	986.8	564.4	685.2	579.9	573.1	425.2	362.2	1519.6	1287.2	1289.5	1149.8	1378.6	1253.7	-1.28
MJ_RS07940	727.1	2822.1	2664.6	989.2	1260.2	723.3	745.2	453.3	411.5	351.3	321.8	706.6	556.0	1.57
MJ_RS07970	986.8	846.6	837.4	758.4	451.7	801.3	689.7	176.3	110.4	117.3	295.8	268.1	196.6	1.94
MJ_RS07975	103.9	407.6	456.8	264.8	259.6	235.0	230.4	110.1	53.4	52.2	109.5	127.7	177.6	1.37
MJ_RS08040	259.7	282.2	266.5	390.8	487.6	380.3	373.0	147.7	109.8	96.4	123.5	116.4	119.6	1.69
MJ_RS08070	51.9	344.9	342.6	230.8	325.1	260.7	248.7	30.9	118.3	193.8	22.6	57.6	41.0	1.77
MJ_RS08085	0.0	0.0	0.0	10.7	4.2	11.6	9.9	28.3	27.8	20.4	120.5	14.1	44.8	-2.26
MJ_RS08090	0.0	62.7	38.1	15.5	20.1	44.0	29.0	73.2	102.3	77.2	320.7	45.3	118.0	-2.11
MJ_RS08095	51.9	31.4	0.0	11.6	10.6	21.6	16.6	42.8	82.2	74.4	162.1	37.6	77.1	-2.32
MJ_RS08110	0.0	0.0	38.1	8.7	6.3	11.6	5.8	22.9	32.5	31.4	66.3	22.1	31.4	-2.01
MJ_RS08115	0.0	0.0	0.0	11.6	9.5	21.6	20.7	48.3	59.9	43.3	108.9	28.2	43.6	-1.90
MJ_RS08130	103.9	188.1	114.2	139.7	112.9	102.1	117.7	300.9	208.6	299.3	383.8	299.4	379.0	-1.37
MJ_RS08135	103.9	62.7	38.1	61.1	81.3	68.1	49.7	231.4	177.9	197.2	167.0	208.5	180.2	-1.58
MJ_RS08140	0.0	0.0	76.1	26.2	26.4	27.4	19.1	70.3	43.4	45.3	76.1	71.5	136.0	-1.57
MJ_RS08175	0.0	62.7	76.1	49.5	52.8	49.8	45.6	32.1	14.4	12.6	26.0	29.5	28.7	1.05
MJ_RS08240	1610.0	689.8	609.1	907.7	1106.1	854.5	792.4	2320.2	2354.8	2807.5	2235.9	1452.4	1047.5	-1.14
MJ_RS08245	103.9	31.4	152.3	42.7	31.7	26.6	19.1	100.8	69.2	68.3	93.3	87.7	149.6	-1.36
MJ_RS08275	0.0	62.7	38.1	12.6	9.5	13.3	15.7	38.1	39.2	34.1	29.1	25.9	25.9	-1.19
MJ_RS08280	0.0	31.4	38.1	15.5	24.3	19.9	17.4	64.4	61.3	51.5	42.9	43.3	50.7	-1.43
MJ_RS09390	103.9	0.0	0.0	27.2	28.5	34.0	37.3	73.0	87.5	68.0	53.7	52.0	57.1	-1.04
MJ_RS08285	51.9	125.4	114.2	98.9	85.5	106.3	98.6	200.7	298.5	251.7	161.4	185.6	191.6	-1.13
MJ_RS08290	0.0	62.7	38.1	31.0	31.7	20.8	28.2	120.5	88.9	77.6	76.8	111.3	134.4	-1.85
MJ_RS08340	207.7	125.4	418.7	93.1	87.6	49.8	39.8	531.3	668.2	787.4	428.9	71.9	285.6	-1.87
MJ_RS08425	155.8	31.4	152.3	143.5	114.0	125.4	146.7	54.1	68.2	49.0	45.6	47.0	52.8	1.30
MJ_RS08540	986.8	1097.5	951.6	825.3	1125.1	701.7	620.0	347.9	549.7	493.8	290.8	326.4	319.6	1.18
MJ_RS08545	779.0	533.1	723.2	890.3	770.5	856.2	825.6	269.8	345.3	299.3	249.2	297.2	291.1	1.46
MJ_RS08550	779.0	564.4	875.5	1014.4	857.0	745.7	794.1	305.6	347.0	232.0	275.4	297.2	245.6	1.54
MJ_RS08585	467.4	470.3	571.0	599.3	485.5	637.8	661.5	257.4	299.5	331.0	275.4	260.1	205.8	1.09
MJ_RS08590	415.5	344.9	532.9	656.6	581.6	686.8	712.9	316.0	271.9	257.7	300.9	344.5	277.6	1.05
MJ_RS08680	0.0	94.1	38.1	25.2	29.6	43.2	54.7	24.7	6.1	5.7	17.1	18.6	21.1	1.35
MJ_RS08725	0.0	62.7	38.1	23.3	20.1	21.6	16.6	255.1	157.5	170.2	141.8	193.0	160.1	-3.09
MJ_RS08730	51.9	62.7	38.1	67.9	58.1	65.6	48.9	327.5	175.5	177.5	212.9	275.1	279.2	-2.02
MJ_RS08785	103.9	0.0	114.2	59.2	49.6	48.2	59.7	146.6	83.2	116.6	128.9	124.5	116.4	-1.12
MJ_RS08900	259.7	188.1	266.5	64.0	50.7	37.4	41.4	208.5	227.3	235.9	188.3	273.4	268.6	-1.41
MJ_RS08995	0.0	156.8	190.3	116.4	128.8	128.7	116.0	44.3	40.4	43.1	43.5	47.4	49.6	1.45
MJ_RS09015	0.0	0.0	76.1	13.6	8.4	27.4	21.6	6.1	4.9	3.9	7.5	8.4	9.2	1.46
MJ_RS09020	103.9	31.4	38.1	147.4	126.7	168.6	122.7	43.7	31.2	29.1	60.8	50.8	60.1	1.46
MJ_RS09025	0.0	31.4	190.3	191.1	127.7	132.0	131.0	60.6	43.0	36.2	71.7	61.9	65.7	1.25
MJ_RS09030	0.0	62.7	0.0	69.8	74.9	57.3	71.3	11.9	13.6	11.9	13.5	15.8	16.0	2.21

MJ_RS09035	0.0	0.0	114.2	18.4	33.8	29.9	26.5	7.2	4.3	5.0	9.6	8.8	13.6	1.79
MJ_RS09040	0.0	0.0	38.1	8.7	9.5	8.3	11.6	3.1	1.2	2.5	3.4	4.0	4.0	1.68
MJ_RS09415	51.9	125.4	0.0	65.0	50.7	94.7	95.3	16.2	37.3	54.3	31.6	29.6	24.2	1.22
MJ_RS09235	571.3	407.6	609.1	540.2	487.6	893.5	940.8	260.9	216.1	217.8	225.0	289.7	309.9	1.39
MJ_RS09240	51.9	0.0	152.3	193.0	130.9	355.4	410.3	78.6	66.6	87.0	78.5	76.3	98.6	1.41
